# Epigenomic variability is associated with age‐specific naïve CD4 T cell response to activation in infants and adolescents

**DOI:** 10.1111/imcb.12628

**Published:** 2023-03-01

**Authors:** Samira Imran, Melanie R Neeland, David J. Martino, Stephen Peng, Jennifer Koplin, Shyamali C Dharmage, Mimi LK Tang, Susan Sawyer, Thanh Dang, Vicki McWilliam, Rachel L Peters, Susan Prescott, Kirsten P Perrett, Boris Novakovic, Richard Saffery

**Affiliations:** ^1^ Murdoch Children's Research Institute, and Department of Paediatrics University of Melbourne, Royal Children's Hospital Flemington Road Parkville VIC Australia; ^2^ Wal‐yan Respiratory Research Centre, Telethon Kids Institute Perth Australia; ^3^ University of Western Australia Perth WA Australia; ^4^ Allergy and Lung Health Unit Melbourne School of Population and Global Health University of Melbourne Melbourne VIC Australia; ^5^ Department of Allergy and Immunology Royal Children's Hospital Melbourne VIC Australia; ^6^ Centre for Adolescent Health Royal Children's Hospital Melbourne Melbourne VIC Australia; ^7^ School of Medicine The University of Western Australia 35 Stirling Highway Crawley WA Australia; ^8^ Telethon Kids Institute 15 Hospital Avenue Nedlands WA Australia; ^9^ Department of Immunology Perth Children's Hospital 15 Hospital Avenue Nedlands WA Australia

**Keywords:** adaptive immunity, adolescents, aging, DNA methylation, infants, nCD4 T cells

## Abstract

Childhood is a critical period of immune development. During this time, naïve CD4 (nCD4) T cells undergo programmed cell differentiation, mediated by epigenetic changes, in response to external stimuli leading to a baseline homeostatic state that may determine lifelong disease risk. However, the ontogeny of epigenetic signatures associated with CD4 T cell activation during key developmental periods are yet to be described. We investigated genome‐wide DNA methylation (DNAm) changes associated with nCD4 T activation following 72 h culture in media+anti‐CD3/CD28 beads in healthy infants (aged 12 months, *n* = 18) and adolescents (aged 10–15 years, *n* = 15). We integrated these data with transcriptomic and cytokine profiling from the same samples. nCD4 T cells from both age groups show similar extensive epigenetic reprogramming following activation, with the majority of genes involved in the T cell receptor signaling pathway associated with differential methylation. Additionally, we identified differentially methylated probes showing age‐specific responses, that is, responses in only infants or adolescents, including within a cluster of T cell receptor (TCR) genes. These encoded several TCR alpha joining (TRAJ), and TCR alpha variable (TRAV) genes. Cytokine data analysis following stimulation revealed enhanced release of IFN‐γ, IL‐2 and IL‐10, in nCD4 T cells from adolescents compared with infants. Overlapping differential methylation and cytokine responses identified four probes potentially underpinning these age‐specific responses. We show that DNAm in nCD4T cells in response to activation is dynamic in infancy and adolescence, with additional evidence for age‐specific effects potentially driving variation in cytokine responses between these ages.

## Graphical abstract

This is the first study to show that nCD4 T cell DNA methylomes from infants and adolescents show age‐specific responses to activation, with specific probes responding in either age group, epigenetic reprogramming at several T cell receptor genes as well as at key immune pathways. These findings contribute to our understanding of the healthy nCD4 T cell response in critical age groups, and establish a baseline reference that may be screened for potential biomarkers in the context of disease.

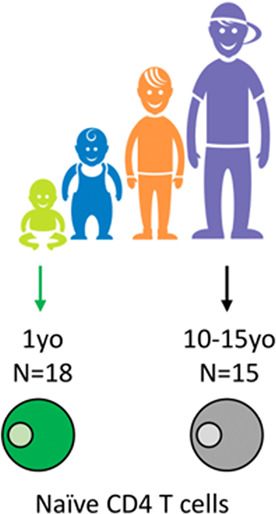

## INTRODUCTION

Aging and functional immunity are inherently interconnected, with the development of the immune system shaped by various environmental factors encountered at different stages of life.^1^, ^2^ This is particularly true in the early postnatal environment (infancy) and with development of a balanced immune response continuing through childhood into adolescence.^3^ As such, infancy and adolescence represent some of the most critical periods for immune development, and improper immune development can result in a number of adverse immune phenotypes.^1^, ^4^, ^5^, ^6^, ^7^

CD4 T cells form an integral part of the adaptive immune response, with naïve CD4T (nCD4T) cells comprising the majority of circulating T cells in infants.^8^ nCD4T cells serve as potent precursors of a number of effector cell types (such as Th1, Th2, Th9 and Th17) playing crucial roles in health and disease.^4^, ^9^ Differentiation into these CD4^+^ T cell subsets is triggered by the T cell receptor signaling pathway following activation by antigens or external stimuli such as cytokine environments.^7^, ^10^, ^11^, ^12^ This plasticity of nCD4T cells is further mediated by a number of epigenetic mechanisms, including DNA methylation (DNAme), which regulate chromatin accessibility at specific sites and subsequent gene expression, thereby dictating T cell fate.^13^ To date, studies examining epigenetic processes associated with T cell development have largely focused on chromatin and histone modifications.^11^, ^14^ DNAm studies assessing changes associated with T cell activation have mainly used targeted approaches focused on selected genes and gene promoter and thus there is limited knowledge of changes in the genome‐wide DNA methylation landscape.^15^, ^16^, ^17^

We previously demonstrated altered T cell activation responses in infants and adolescents with food allergy relative to non‐food allergic controls.^6^, ^18^ However, a direct comparison of epigenetic remodeling during T cell activation in healthy infants and adolescents is yet to be described. This is important as infancy and puberty are both critical windows that initiate the maturation of several systems of the human body, including the immune system.^3^ A signature for a healthy T cell response represents a baseline profile of a balanced immune response which may be used as a reference to compare aberrant T cell activation patterns across various immune disorders.

In this study, we characterized age‐linked epigenomic patterns underpinning responses to activation in nCD4 T cells by comparing DNA methylation patterns in infants and adolescents.

## RESULTS

### DNAm variation associated with nCD4T cell activation in both infants and adolescents

**Figure 1:**
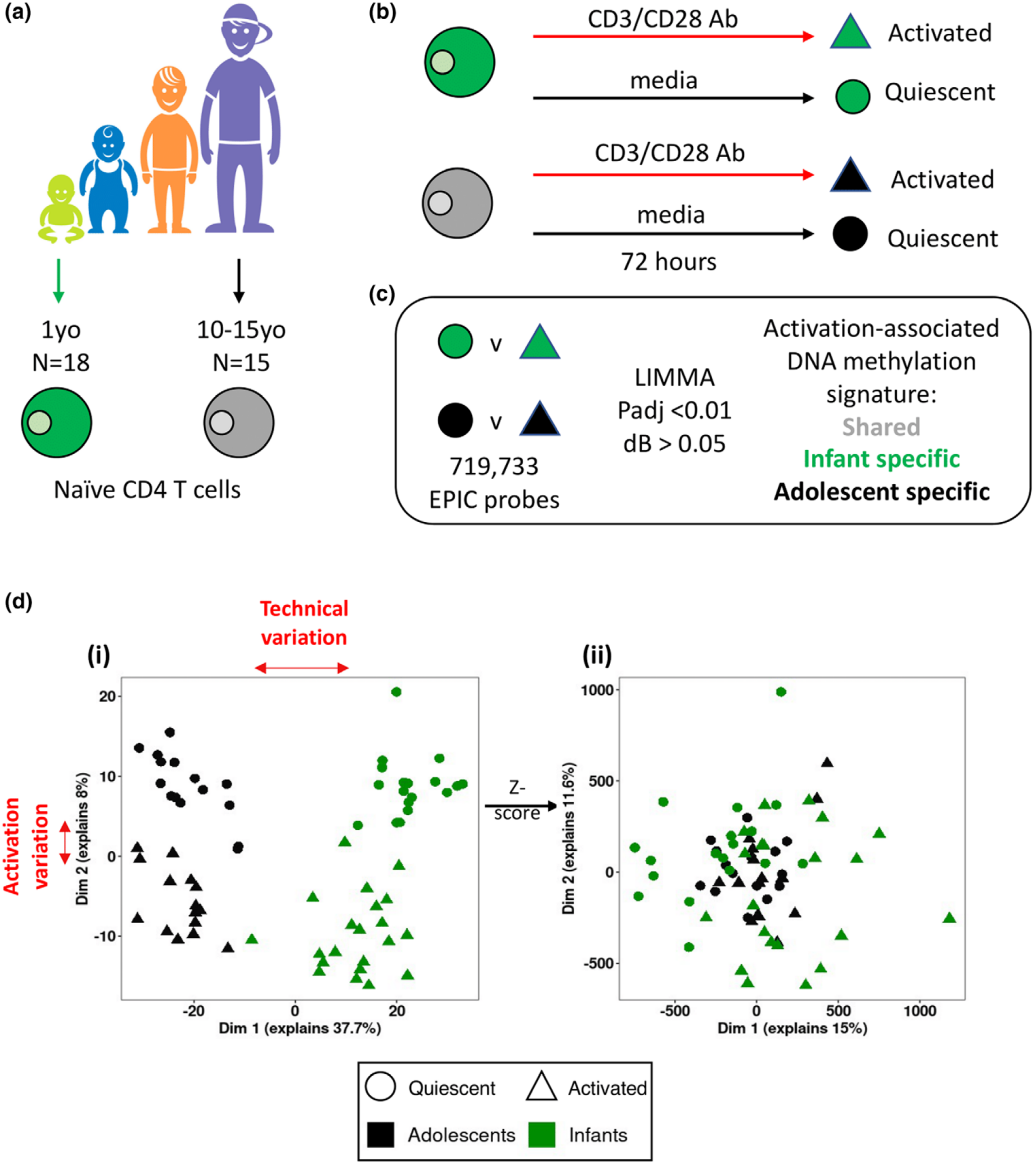
**(a)** Summary of individuals in age groups assessed in this study; data were derived from naïve CD4 T cells isolated from infants (*n* = 18, shown in green) and adolescents (*n* = 15, shown in gray). **(b)** Study design of nCD4 T cell culture in infants and adolescents. **(c)** Analysis approach for DNA methylation in nCD4 T cells across age groups. Activated and quiescent nCD4T cell epigenomes were compared in infants and adolescents, using cutoffs of adjusted *P*‐value < 0.01, and *Δβ* > 5%, and sets of probes showing age specific responses were identified. **(d) (i)** Principal components analysis (PCA) showing technical variation between infant and adolescent datasets **(ii)** PCA showing minimal variation between infant and adolescent datasets post‐scaling.

DNA methylation profiling was carried out on isolated naïve CD4 T cell genomic DNA following 72 h culture in either media alone, or with anti‐CD3/CD28 beads, using the widely validated Infinium MethylationEPIC arrays. We assessed DNA methylation data from 18 infants (GSE189148) and 15 adolescents (GSE114135) and found a total of 719 733 probes remaining across both datasets following initial pre‐processing steps. The overall study design is outlined in Figure 1a–c and Table 1.

**Table 1:** Table of demographics for subsets of HealthNuts and SchoolNuts cohorts used in this study.

	Infants	Adolescents
Total number	18	15
Sex: male, *n* (%)	9 (50%)	9 (60%)
Age at blood collection median (min–max)	13 (10–15) months	13 (11–14) years
Current eczema, *n* (%)	4 (22%)	7 (47%)

We carried out a principal components analysis (PCA) of *β*‐values of all 719 733 probes common to both datasets (infants and adolescents, including both quiescent and activated samples). This indicated a clear difference between activated and quiescent samples (Dim 2), but also revealed likely major technical differences between the two datasets (Dim 1) (Figure 1d(i)). As such, *β*‐values were scaled within each dataset prior to repeating PCA, which removed this technical effect (Figure 1d(ii)).

In total, 19 415 activation‐associated DMPs were identified in infants, with an adjusted *P*‐value < 0.01 and a methylation difference (*Δβ*) greater than 5% between activated and quiescent samples (Table 2). Of these, 5014 DMPs gained methylation (hypermethylated) and 14 401 DMPs lost methylation (hypomethylated) following activation (Supplementary table 1).

**Table 2:** Summary of differentially methylated probes between quiescent and activated comparisons in infants and adolescents

	Infants	Adolescents
DMPs reaching *P*‐value significance		
Gain in methylation	5014	3072
Loss in methylation	14 401	4728
Total probes	19 415	7800

In adolescents, 7800 activation‐associated DMPs were identified (adjusted *P*‐value < 0.01 and a *Δβ* > 5%) (Table 2). Among these DMPs, 3072 showed a gain in methylation following activation, while 4728 DMPs showed a loss in methylation following activation (Supplementary table 1).

**Figure 2:**
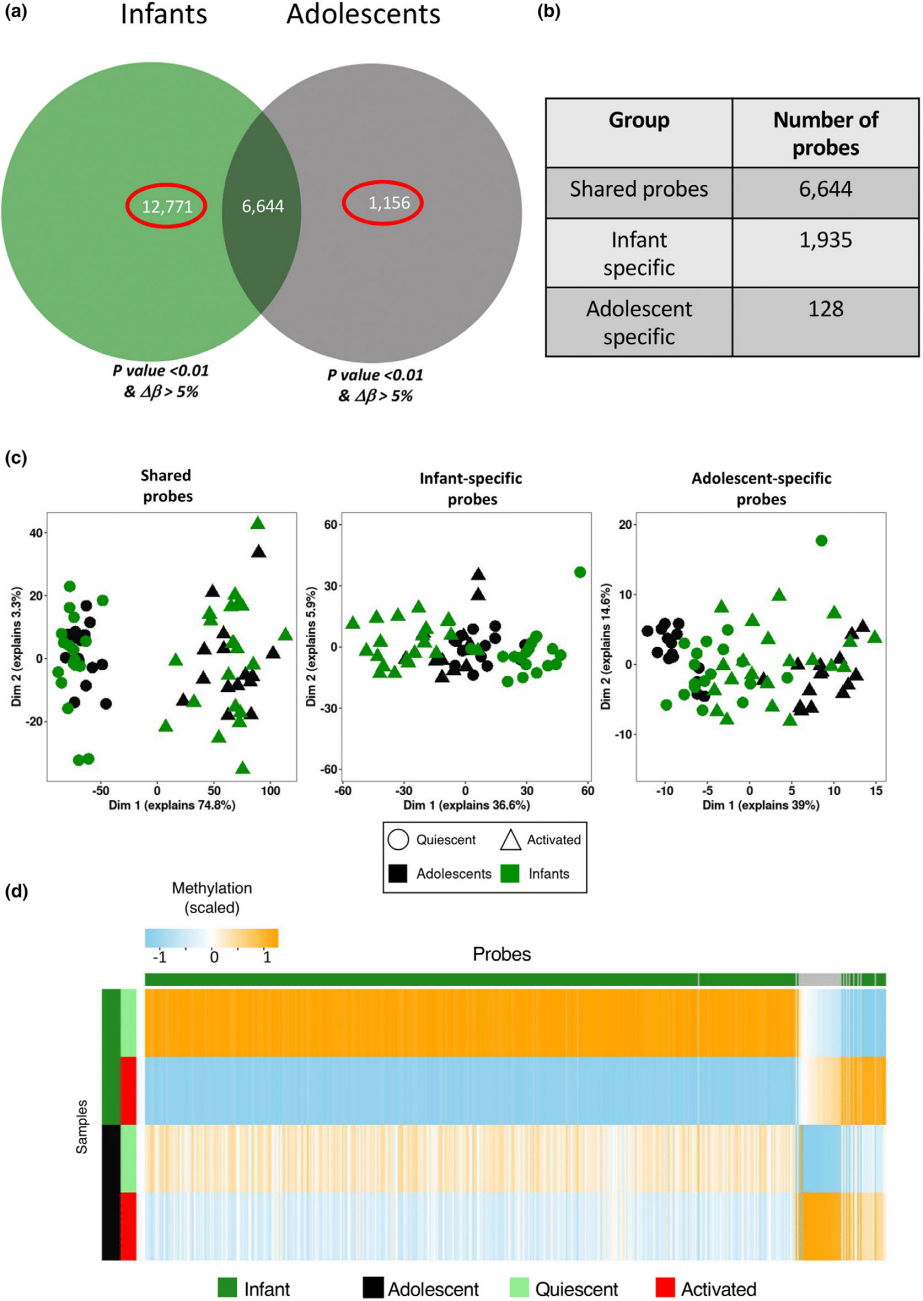
**(a)** Venn diagram showing extent of overlap between probes showing a change in methylation with activation between infants and adolescents, reaching analysis threshold of *P*‐value < 0.01 and/or *Δβ* > 5%. **(b)** Summary of probes showing shared patterns of activation in infants and adolescents, as well as probes showing age‐specific responses. **(c)** PCA of probes showing shared patterns of activation, infant specific responses and adolescent specific responses. **(d)** Heatmap of probes showing infant specific responses and adolescent specific responses, showing that the majority of infant‐specific probes show loss in methylation with activation.

Overall, 6644 DMPs showed shared patterns of methylation in response to activation across infants and adolescents. This set of probes was classified as “shared. CpGs” across infants and adolescents (Figure 2a). One of these shared probes (cg07355000) showed opposing direction of effect between infants and adolescents. This probe was omitted from the set of shared probes in further analysis, and was assessed separately (Supplementary figure 2).

### Age‐specific DNAm variation associated with nCD4T cell activation

To identify age‐specific responses to activation, we identified probes exclusively reaching a *P*‐value (< 0.01) and *Δβ* significance (> 5%) in comparisons of quiescent and activated samples in either infants or adolescents. This revealed 12 771 DMPs specific to infants, with far fewer (1156) in adolescents (Figure 2a). Despite failing to reach the *P*‐value significance threshold, many of these probes continued to show a considerable change in methylation in the “non‐responsive” age group. As such, an effect size cutoff of *Δβ* < 2% was implemented to identify truly unresponsive probes among these sets of DMPs showing age‐specific patterns of methylation. This reduced the numbers of DMPs to 1935 DMPs exclusively changing in response to activation in infants, and 128 DMPs exclusively changing in response to activation in adolescents (Figure 2b, Supplementary table 2).

Principal components analysis was performed on each of these sets of probes (shared, infant‐specific and adolescent‐specific) using the compiled dataset consisting of scaled beta values of infant and adolescent samples (Figure 2c). These PCA plots show clear separation of quiescent and activated samples in both infants and adolescent data based on the shared CpGs. A PCA of the infant‐specific DMPs highlighted the separation of the infant, but not adolescent samples into two groups, while a plot of the adolescent‐specific DMPs showed the reverse effect as anticipated.

Methylation values of these selected probes in quiescent and activated samples were plotted as boxplots in each age group, which further demonstrate the age‐specific nature in these probes (Supplementary figure 2). Probes specific to infants showed an impaired response in adolescents, and *vice versa*. A heatmap of age‐specific DMPs shows that the majority of the infant‐specific DMPs lose methylation (1831/1935), whereas the majority of adolescent‐specific DMPs gain methylation (120/128) following activation (Figure 2d).

### Functional enrichment analysis of gene‐associated, age‐specific DMPs

To determine the functional relevance of the identified DMPs, we carried out functional enrichment analysis on genes in the vicinity of these probes and assessed the genomic context of these probes relative to gene transcription start sites, CpG islands and open chromatin (ATAC‐seq) regions in CD4 T cells (Supplementary figure 3a, b).

**Figure 3:**
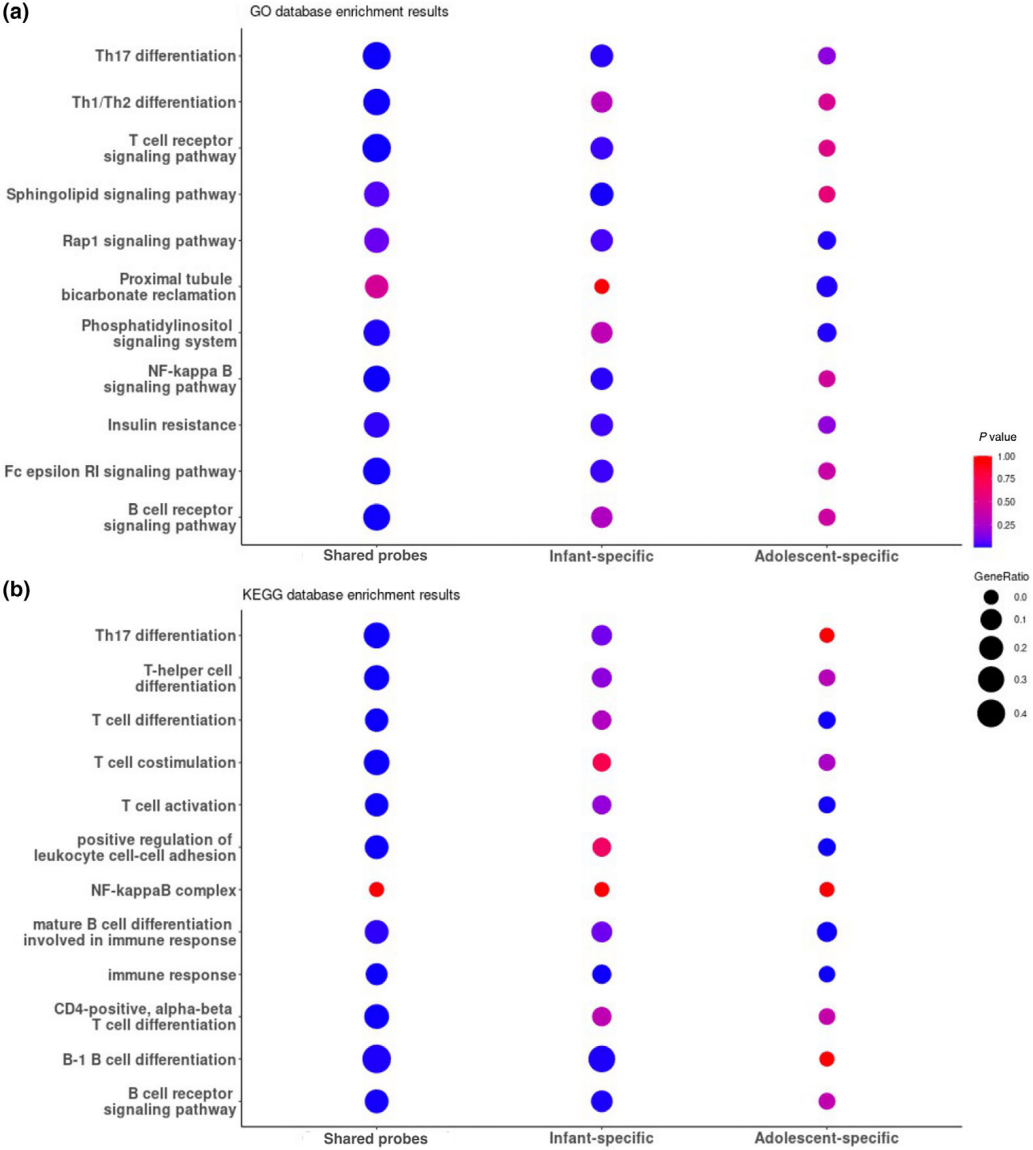
Comparison of functional enrichment terms across genes linked to probes shared patterns of activation, infant specific responses and adolescent specific responses in **(a)** GO:biological process database and **(b)** KEGG database. The size of the bubble plot represents the gene ratio (number of genes linked to DMPs from this study/number of genes in background set); while color represents *P*‐value.

Based on results from both the GO and KEGG database analyses, the set of genes annotated to shared probes were significantly enriched in the T cell receptor signaling pathway (*P*‐value = 3.2 e‐09), were associated with shared DMPs, with 42/74 genes in the pathway showing differential methylation upon activation. Genes associated with the TCR response in infants were enriched for the sphingolipid (*P*‐value = 0.002) and Fc epsilon RI signaling pathways (*P*‐value = 0.011) and insulin resistance (*P*‐value = 0.026) in the KEGG database (Figure 3a); and NF‐kappaB complex (*P*‐value = 0.002), B cell receptor signaling pathway (*P*‐value = 0.004) and B1 B cell differentiation (*P*‐value = 0.005), based on the GO database (Supplementary table 2, Figure 3b). On the other hand, adolescent‐specific DMPs were enriched for phosphatidylinositol signaling system (*P*‐value = 0.007), proximal tubule bicarbonate reclamation (*P*‐value = 0.006) and the Rap1 signaling pathway (*P*‐value = 0.007) in the KEGG database (Figure 3a); and positive regulation of leukocyte cell–cell adhesion (*P*‐value = 5.01 e‐5), mature B cell differentiation involved in immune response (*P*‐value = 2.15 e‐4), and T cell differentiation (*P*‐value = 5.25 e‐4) in the GO database (Supplementary table 2, Figure 3b).

More adolescent‐specific DMPs were located at CpG islands and shores compared with the shared or infant‐specific DMPs (Supplementary figure 3). Genes with most DMPs in their proximity are shown in Supplementary figure 3d. The *DAD1* gene had the highest number of shared DMPs in its proximity, and also a number of infant‐specific and adolescent‐specific DMPs^19^ (Supplementary figure 3d). A closer look at this region reveals a cluster of several genes encoding T cell receptor alpha joining (*TRAJ*) and T cell receptor alpha variable (*TRAV*) genes. The probe showing opposing methylation trends in infants and adolescents (cg07355000) was also located near a *TRAJ* gene (*TRAJ54*) (Supplementary figure 2b).

Comparison of the distribution of these probes relative to gene transcription start sites and open (ATAC‐seq) and dynamic chromatin regions did not suggest notable differences among the probe sets, with the shared, infant‐specific and adolescent‐specific DMPs showing similar trends in distribution. However, among the shared DMPs following activation, those gaining methylation are associated with decreased chromatin accessibility, whereas probes losing methylation show enhanced chromatin accessibility in nCD4T cells following activation, as expected (*Χ* ^2^ (1, *N* = 6643) = 11.43, *P*‐value < 0.01) (Supplementary figure 3c). This suggests that the DNA methylation signatures reported in our study represent dynamic genomic regions serving functional roles in T cell activation.

### DMRs associated with age‐specific T cell activation probes

**Figure 4:**
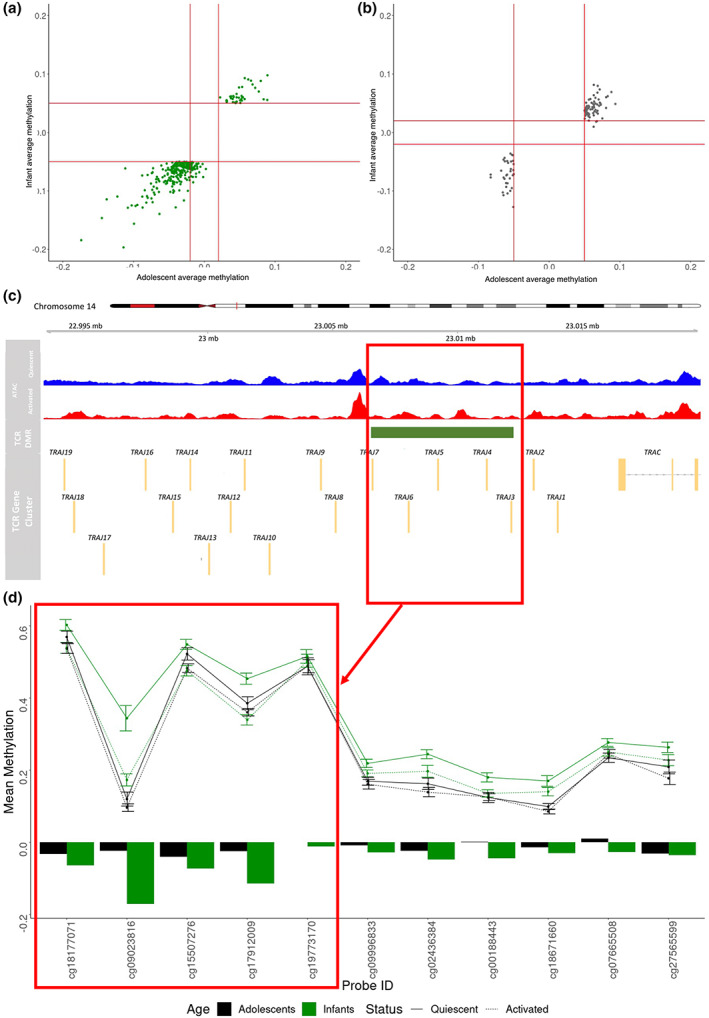
**(a)** Scatterplot of average methylation values of DMRs identified between activated and quiescent samples in infants, plotted against average methylation values of these DMRs in adolescents. Lines represent cutoff values for methylation difference (5% in infants, 2% in adolescents). **(b)** Scatterplot of average methylation values of DMRs identified between activated and quiescent samples in adolescents, plotted against average methylation values of these DMRs in infants. Lines represent cutoff values for methylation difference (5% in adolescents, 2% in infants). **(c)** Location of the *TCR* DMR relative to *TCR* genes. Location of DMR probes on the chr14 chromosome (p arm) and proximity to nearest TCR genes. Overlaid with ATAC‐seq data from quiescent and activated healthy naïve CD4T cells indicate regions of open chromatin. **(d)** Mean methylation values (β values) of probes in infants and adolescents at quiescence and post‐activation within DMR. Error bars represent 95% confidence intervals.

We next identified differentially methylated regions (DMRs) showing a significant change in methylation with activation in infants and adolescents, applying a cutoff of adjusted *P*‐value < 0.01, consisting of > 3 probes showing an average *Δβ* > 5% across all probes in the region. We found a total of 447 DMRs between quiescent and activated samples in infants, where 58 DMRs were hypermethylated following activation, and 389 DMRs were hypomethylated following activation (Supplementary table 3). In adolescents, we found a total of 269 DMRs changing with activation, where 182 DMRs showed a loss in methylation with activation, and 87 DMRs showed a gain in methylation following activation (Supplementary table 3). Of the total 547 DMRs changing with activation, 169 were common to both infants and adolescents, while 34 and 3 DMRs showed infant‐specific and adolescent‐specific activation patterns, respectively (Figure 4a, b).

The majority of these DMRs were associated with open chromatin regions (Supplementary figure 4c, d). However, we found limited overlap of DMRs with regions showing a significant change in chromatin accessibility (*P*‐value < 0.05, fold change > 1.5). An infant‐specific activation DMR of interest was located within the cluster of TCR genes and within an ATAC peak. This DMR consisted of six probes showing a loss in methylation with activation in infant samples, but limited differences between quiescent and activated samples in adolescents (Figure 4c, d).

### Elevated cytokine responses in adolescent nCD4T cells following activation

To determine whether these differences were reflected at the protein level, we used previously published data documenting cytokine concentrations of these samples at quiescence and post‐activation. We also obtained transcriptomic data from the infants at both quiescent and activated conditions, and from activated adolescents, and these were used to corroborate the cytokine data.

**Figure 5:**
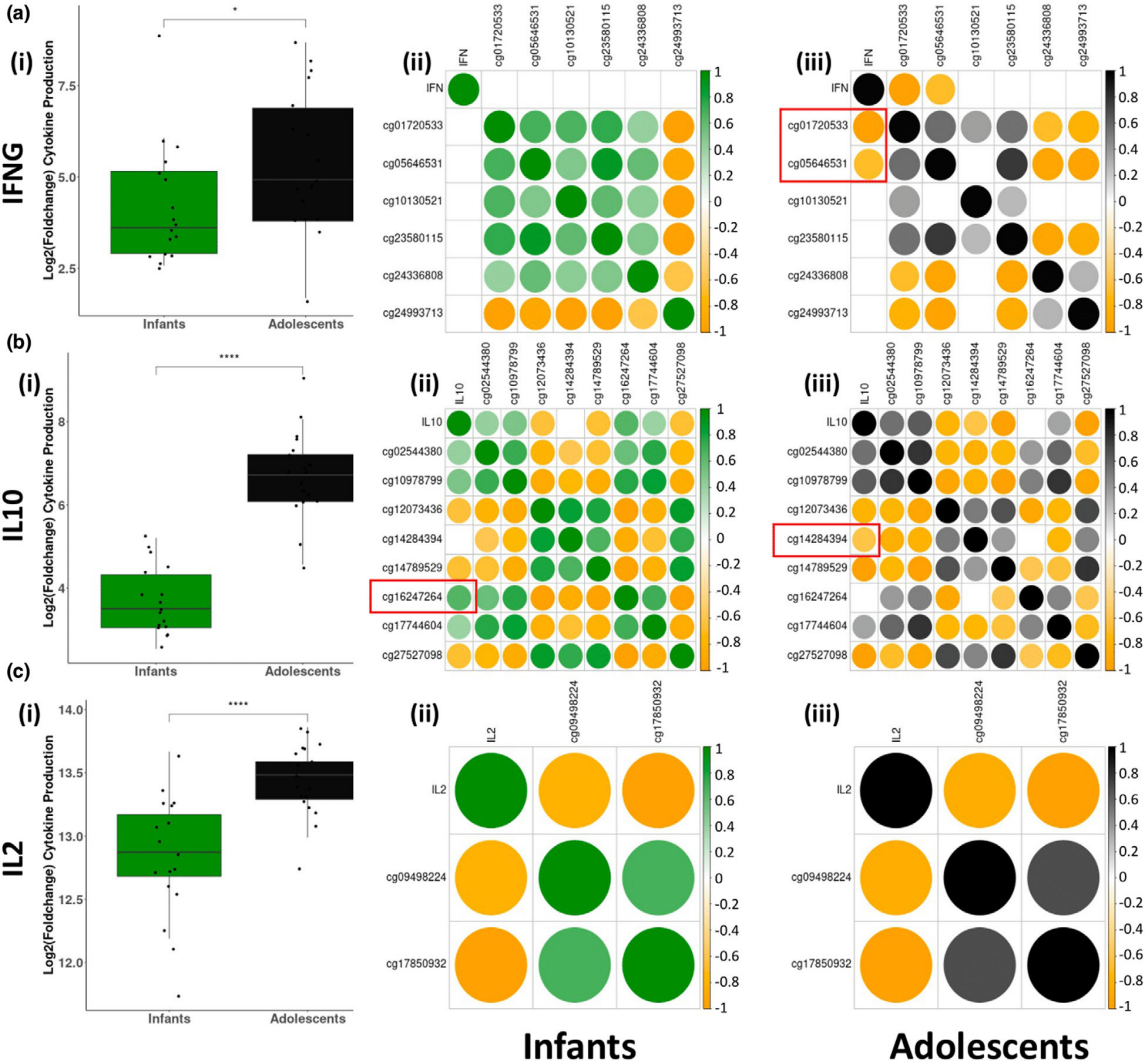
**(a–c)** **(i)** Boxplots of log_2_(fold‐change) of cytokine production between quiescent and activated samples in infants and adolescents. **(ii)** Correlation plots representing Pearson's correlation coefficients of cytokine levels and methylation of age‐specific DMPs near gene encoding cytokine in infant samples, only showing correlations reaching *P*‐value < 0.05. Colors represent magnitude and direction of correlation. **(iii)** Correlation plots representing Pearson's correlation coefficients of cytokine levels and methylation of age‐specific DMPs near gene encoding cytokine in adolescent samples, only showing correlations reaching *P*‐value < 0.05. Colors represent magnitude and direction of correlation. Probes showing age‐specific correlations are shown in red boxes.

There was a significant increase in the levels of all these cytokines (IL2, IL6, IL10, IFN‐γ, TNF) following activation in both infants and adolescents (Supplementary tables 4 and 5). Comparison of the fold changes of these cytokines between quiescent and activated samples shows that nCD4 T cells from adolescents produce significantly higher levels of IL2, IL10 and IFN‐γ in response to activation, relative to infants (Figure 5). However, there were minimal differences in the production of IL‐6 and TNF‐α between infants and adolescents (Supplementary figure 5). Protein expression of these cytokines was also reflected at the gene expression level in infants and adolescents for *IL2, IL6, IL10* and *IFNG*, but there was no evidence for expression of *TNF* in infant or adolescent samples (Supplementary figure 5).

Next, we assessed the correlation between these datasets, by compiling matched epigenomic and proteomic data in either age group. We scanned for DMPs changing with activation (all DMPs showing *P*‐value < 0.01 and *Δβ* > 5% between activated and quiescent samples, regardless of age‐specific responses) located within 1 Mbp of the transcription start sites of genes encoding the selected cytokines and correlated their cytokine levels with methylation levels of these DMPs. Among cytokines showing age‐related variation, IFN‐γ production was significantly correlated with two DMPs in adolescents (cg01720533 and cg05646531) (Figure 5a), and IL‐10 production was significantly correlated with cg16247264 in infants and cg14284394 in adolescents (Figure 5b).

We also found ATAC‐seq peaks within 1Mbp of *IL2, IL6, IFN‐γ* and *IL10*, but no ATAC peaks were found near *TNF* (Supplementary table 1). Only *IL*6 was associated with an ATAC peak showing differential accessibility between quiescent and activated samples.

### Regulation of T cell activation pathway

**Figure 6:**
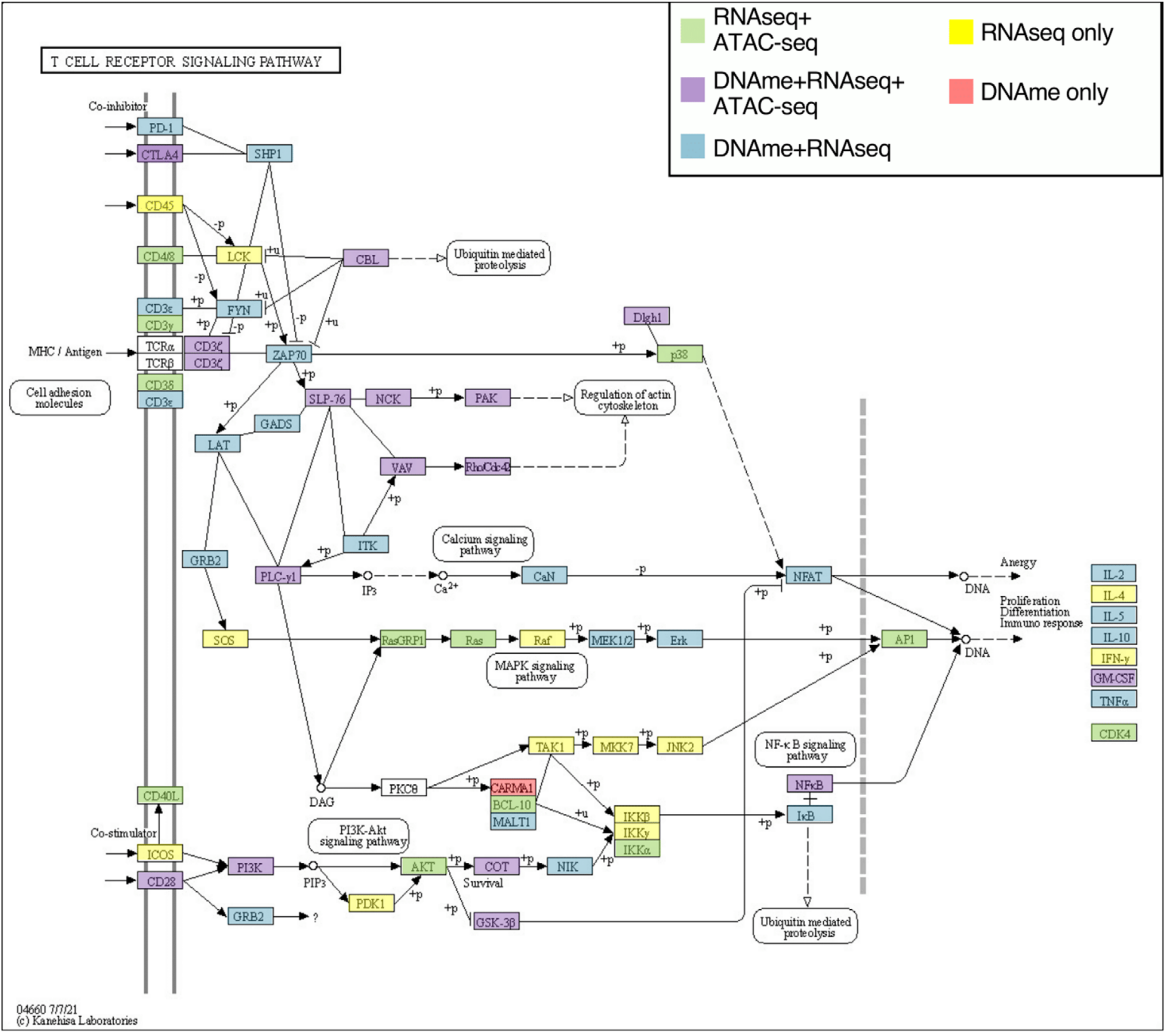
KEGG diagram of T cell receptor signaling pathway, colored by various levels of regulation in infants: DNA methylation, gene expression and/or chromatin accessibility.

Next, we sought to determine the degree to which genes within the T cell activation pathway are regulated by coordinated epigenetic remodeling. To do this, we compiled DMPs, differential ATAC‐seq peaks and differentially expressed genes between quiescent and activated nCD4T cells from infants, where we had data for both quiescent and activated cells. Using the pathview package in R, we highlighted the T cell receptor signaling pathway based on these data. Of the 74 genes in this pathway, 22 genes were associated with changes in DNA methylation, gene expression and chromatin accessibility; 19 genes showed differential DNA methylation and gene expression; 10 genes showed differential gene expression and chromatin accessibility; 12 genes only showed differential gene expression; and a single gene, *CARMA1*, was exclusively associated with changes in DNA methylation (Figure 6).

### Overlaps with previous studies assessing age‐associated T cell methylation patterns

Several studies have previously identified age‐associated epigenetic and transcriptional differences in CD4 T cells.^20^, ^21^, ^22^ Specifically, we compared our data with a study by Dozmorov *et al*. (2018) that profiled aging CD4 T cell epigenomes, uncovering 11 431 CpG sites showing age‐related changes.^20^ Based on this analysis, 146 of our age‐specific activation response DMPs were associated with the aging CpG signature outlined in this study (Supplementary table 1). Functional enrichment analysis carried out on the genes linked to these probes revealed enrichment for several immune pathways such as the NF‐κβ signaling pathway, the T cell receptor signaling pathway and the TNF signaling pathway (Supplementary table 5).

## DISCUSSION

Our study profiled epigenomic changes in naïve CD4 T cells in response to activation across two critical periods of immune development: infancy and adolescence. We identified infant‐specific and adolescent‐specific DNA methylation changes in response to activation. These data were correlated with proteomic data from the same individuals to establish age‐specific signatures for nCD4T cell activation across multi‐omic datasets. Our findings contribute to the body of literature profiling changes in the T cell epigenetic landscape in healthy individuals in response to activation, which will prove invaluable as a reference for aberrant activation patterns of nCD4 T cells in various diseases.^5^, ^6^, ^7^, ^11^ Moreover, many diseases in adults have origins in childhood, therefore building a reference for healthy T cell responses in early age groups will help to establish a baseline pattern for T cell responses that can be screened for potential disease biomarkers.

Our data indicate that the T cell methylome is extensively remodeled in response to activation, irrespective of age, with most genes in the T cell‐specific pathways (T cell receptor signaling and T cell differentiation) associated with activation‐dependent DMPs. Previously, using the same non‐food allergic infant DNA methylation data, we found that naive CD4T cells from infants with egg allergy exhibit diminished lymphoproliferative responses following non‐specific activation with anti‐CD3 and anti‐CD28, reflected at the molecular level by impaired expression of T cell activation genes, and epigenetic remodeling at genes linked to metabolic pathways.^6^ Overall, activation‐dependent DMPs showed an inverse relationship with chromatin accessibility, with an increase in DNA methylation associated with loss of accessibility, and *vice versa*.^11^ Genes in the TCR signaling pathway showed coordinated DNA methylation, gene expression and open chromatin dynamics, with no gene only associated with changes in chromatin accessibility. This indicates that when nucleosome positioning changes in response to T cell activation, it also involves DNA methylation and gene expression changes.

The methylome is dynamic throughout the human lifespan, and in this study, we demonstrate that such variation is apparent in immune cell responses as well, by highlighting CpG sites that show age‐specific methylation changes during T cell activation in infants and adolescents. Overall, we found that probes showing adolescent‐specific responses largely showed a gain in methylation following activation, in contrast to the probes showing infant‐specific responses. Studies have previously shown that genome‐wide hypomethylation is associated with aging, whereas gene promoter regions exhibit increasing methylation with age.^23^, ^24^ While we did not find any differences in the distribution of these age‐specific DMPs relative to transcription start sites, this trend suggests that these age‐specific activation signatures may be occurring at functionally relevant sites.

The most prominent age‐specific differences in nCD4 T cell activation were observed at probes located near genes encoding various T cell receptor genes, including a DMR responding specifically in infants and not adolescents at this locus, as well as a probe showing opposing trends in methylation between infants and adolescents. The TCR repertoire undergoes several functional changes across the lifecourse, which includes changes in “publicity”, and a decrease in TCR repertoire diversity with age as a result of thymic involution resulting in decreased generation of nCD4T cells.^25^, ^26^ However, these past studies have mainly been carried out at the transcriptional and proteomic level, and therefore epigenetic signatures driving these phenotypes have not been investigated. Although we found limited overlaps between our data and previous studies, the enrichment for key immune pathways among the genes associated with these CpG sites indicates major age‐associated changes in these responses.^20^ This highlights the need to establish more updated aging signatures based on newer microarray approaches, or potentially identify cell‐specific signatures for aging.^27^, ^28^

Future studies integrating in‐depth TCR analysis with DNAme data will prove useful in establishing the relationship between DNA methylation and age‐related variation in TCR function.

The elevated cytokine output observed in adolescents in our data is in line with previous studies showing increased cytokine production with age.^29^, ^30^, ^31^, ^32^, ^33^ This augmented response is postulated to compensate for age‐related decline in immune function.^31^ For instance, Haynes *et al*. showed that the introduction of inflammatory cytokines are capable of restoring impaired responses in CD4T cells showing suboptimal proliferation and cytokine production following immunization with Ag + alum.^31^ Another study found significantly higher levels of IFN‐γ and IL4 in activated nCD4T cells from elderly individuals, compared with young adults.^33^ Our group has previously reported increased production of IL5, IL9, IL13 and IL22 between activated nCD4 T cells from infants compared to neonates, but did not find any epigenetic variation between the two age groups.^29^ We are therefore the first to identify potential CpG sites that may be associated with this altered response. Further study is required to validate these results and to determine the relevance of these CpG sites across multiple age groups.

Our study presents crucial information regarding epigenetic regulation of T cell activation in infants and adolescents. However, we recognize the limitations in our datasets. We acknowledge the small sample sizes of the age groups in this study, and the fact that the infant and adolescent methylation datasets had been generated at different timepoints, as well as variability between individuals within age groups. We were thus unable to track changes in T cell responses in the same individuals over time. Nevertheless, the spread in our data allows the data to be generalized across age groups and to characterize collective responses in age groups, and we incorporated data from all available covariates into the linear regression model for differential methylation analysis. We did not have consistent data for quiescent and activated T cells across all samples for the transcriptomic and chromatin accessibility among the adolescents. Future studies should incorporate multi‐omic data from all stimulation conditions in the same individuals over time to obtain a clear pictures of epigenetic mechanisms regulating T cell activation across different age groups. We also recognize that adolescence is marked by the onset of puberty, which may result in variable responses according to pubertal maturation and between males and females. However, our sample size did not allow us to stratify the analyses by sex. An investigation of sex‐related changes in age‐specific T cell responses will provide further insight into how puberty differentially affects immune development by sex.

## CONCLUSION

In conclusion, this is the first study to show that nCD4 T cell DNA methylomes from infants and adolescents show age‐specific responses to activation, with specific probes responding in either age group, epigenetic reprogramming at several T cell receptor genes as well as at key immune pathways. We also uncovered age‐specific activation‐dependent DMPs correlating with cytokine levels across infants and adolescents that may be associated with enhanced cytokine production with age. Further study is warranted to validate these findings across additional age groups, as well as to track these changes within the same individuals over time and integrate these data with detailed immune profiling and proteomic analyses.

## METHODS

### Clinical samples and data collection

This study was carried out using peripheral blood mononuclear cells (PBMC) samples from a subset of non‐food allergic infants enrolled in the HealthNuts cohort, aged between 13 and 19 months at baseline (mean age 15 months), and a subset of non‐food allergic adolescents enrolled in the SchoolNuts study (mean age 13 years). Both the HealthNuts and SchoolNuts studies were established to identify the prevalence of food allergy in adolescents aged between 10 and 15 years. The demographics for the individuals assessed in this study are outlined in Table 2. The non‐food allergic individuals had a history of tolerating the food.

### Naïve CD4 T cell isolation and activation

#### Isolation of nCD4T cell populations *via flow cytometry and culture*

As described previously, PBMCs were isolated from blood samples using Ficoll‐Paque density gradient centrifugation and cryopreserved in liquid nitrogen.^34^ PBMCs were thawed (mean viability based on trypan blue exclusion: 93%) and total nCD4T cell populations (CD3^+^ CD4^+^CD45RA^+^ CCR7^+^) were isolated using fluorescence activated cell sorting (BD FACS‐ARIA Fusion cell sorter).

Isolated naïve CD4 T cells were resuspended at 8 × 10^4^ per 200 μL in T‐cell activation media (RPMI supplemented with 10% FCS, penicillin streptomycin and 200 IU mL^–^^1^ of IL2) and seeded (8 × 10^4^/well) for 72 h at 37°C, 5% CO_2_. At least 1.6 × 10^5^ cells (duplicate wells) were cultured in RPMI alone (quiescent samples), while another 1.6 × 10^5^ cells were stimulated with anti‐CD3/CD28 T‐cell activator Dynabeads (activated samples). Following this 72 h incubation, replicate wells were pooled and centrifuged to obtain cell pellets. Cell counts of samples in media alone and following activation showed similar cell proliferation rates across infants (fold change = 2.9) and adolescents (fold change = 3.1). Supernatants were aspirated and stored separately at −30°C for cytokine analysis, and cell pellets were resuspended in 350 μL of RLT + 2ME (QIAGEN) and stored at −80°C for later DNA and RNA extractions.

#### Isolation of nucleic acids

DNA and RNA were extracted using the QIAGEN AllPrep DNA/RNA micro‐kit, according to manufacturers’ protocols and quantified on the Qubit fluorometer using the Qubit dsDNA High Sensitivity (HS) assay kit, and the Qubit RNA HS assay kit (Thermofisher Scientific) respectively.

### Infant and adolescent DNA methylation data analysis

Genomic DNA from paired activated and quiescent samples (200–500 ng) from 18 infants and 15 adolescents were randomized in 96 well plates and sent to HuGe‐F (Erasmus MC, Rotterdam, Netherlands) for bisulfite treatment and genome‐wide methylation analysis using Illumina InfiniumMethylationEPIC BeadChips (the EPIC array). The EPIC array is capable of detecting methylation at > 850 000 CpG sites (EPIC probes) across gene bodies, promoters, regulatory elements (ENCODE open chromatin and enhancers).^35^ Briefly, steps for the EPIC array involve quality control, where samples are quantified and quality is assessed to ensure the DNA reaches minimum requirements for the EPIC array. Once the samples pass quality control, they undergo bisulfite treatment to facilitate conversion of unmethylated cytosines to uracil, followed by whole genome amplification (WGA), during which the uracil bases were converted to thymine. Enzymatic cleavage is then carried out to remove dNTPs, primers and enzymes. The samples are then hybridized to the array, and single base extension is performed based on the bisulfite‐converted DNA. Finally, the DNA is stained fluorescently and the chip is scanned using the Illumina iScan System.

#### Data pre‐processing

Raw data from previous studies were available as .iDAT files (GSE114135 and GSE189148).^6^ Due to high technical variability between the datasets (i.e. large batch effect, as a result of samples being run on two separate occasions), data from the infants and adolescents were analyzed separately with the same pipeline. Raw data were pre‐processed using the minfi and MissMethyl packages (available from Bioconductor) in the R statistical environment (v3.6.1).^36^, ^37^

Assessment of sample quality revealed that all samples in the adolescent and infant datasets had a mean detection *P*‐value < 0.01, allowing for all samples to be included in subsequent analysis. The SWAN (Subset‐quantile Within Array Normalization) approach was used to normalize data for technical variation between and within arrays.^38^ Probes were then filtered to remove those with poor average quality scores (*P*‐value > 0.01), cross‐reactive probes and probes associated with single nucleotide polymorphisms (SNPs).^35^ Samples from the adolescent study had been run on two separate batches. To remove any variation resulting from batch effects within age groups we used RUVfit to identify all batch‐affected probes and removed these from analysis from both infant and adolescent data.^39^

Following initial pre‐processing steps, the infant dataset comprised 724 373 probes, while the adolescent dataset 721 171 probes. In order to minimize technical variation, analysis was limited to probes that were present in both datasets (719733).

### Statistical analysis

Differential analysis was carried out on the 719 733 probes using a linear regression model with limma.^40^ Covariates included in linear regression model for infant DNAme data included sex, ancestry, SEIFA quintile, attendance at childcare and sample position on EPIC array (batch, plate well and position on chip). Covariates included in linear regression model for adolescent DNAme data were age, sex, hayfever, wheeze, parent country of birth (within Australia/overseas) and sample position on the EPIC array (batch, plate well and position on chip). Differentially methylated probes in the quiescent *vs* activated comparisons were identified as those showing an FDR‐adjusted *P*‐value < 0.01 and a methylation difference (*Δβ*) > 5%.

The DMRCate tool was used to determine differentially methylated regions (DMRs), and individual probes within these DMRs were identified using Bedtools.^41^, ^42^ Significant DMRs were identified as those showing an FDR‐adjusted *P*‐value < 0.01, and consisting of four or more probes, with an average *Δβ* > 5% across all probes. The nearest genes (within 1 Mb of the transcription start site in any one direction) to DMRs/DMPs were determined using the web‐based GREAT tool.^19^ Functional enrichment analysis on genes linked to these probes was carried out using the GoMeth feature of the MissMethyl package on R.

Beta‐values of the 719 733 probes were scaled within each age group for combined analysis of the two age groups.

### Transcriptomic data analysis

RNA samples from both the infants and adolescents had been prepared using the same approach (Illumina TruSeq Stranded mRNA kit with a starting input of 100 ng) and sequenced on the Illumina NovaSeq 6000 (generating approximately 20 million reads per sample, 100 bp paired‐end) at the Translational Genomics Unit at the Victorian Clinical Genetics Services. Raw fastq files were available for the infant samples at both quiescence and following activation, and for the adolescent samples following activation, as RNA concentrations from quiescent adolescent samples did not reach thresholds for minimum starting input for library preparation and RNA:seq analysis. These were aligned to the human transcriptome (GrCh37 v70) using bowtie2, and gene counts were derived using HTSeq.^43^, ^44^ Non‐protein coding genes and genes exhibiting low expression (average counts per million < 1) were removed from analysis. The limma‐voom pipeline was used to normalize count data and to obtain expression estimates for genes across samples.^45^

### Publicly available open chromatin signatures (ATAC‐seq) data analysis

We accessed publicly available ATAC‐seq data for nCD4 T cell (at quiescence and following activation using an identical protocol) from healthy infants (GSE157174) and bwa was used to align ATAC‐seq reads to human genome assembly hg19.^11^, ^46^ Low quality reads (mapping < 15) were filtered from resulting BAM files using samtools and peaks were called using MACS2.^47^, ^48^ Peaks from all samples were merged into a single ATAC peaks .bed file and reads per peak were counted using bedtools.^42^ bamCoverage from the deeptools package was used to convert bam files into bigwig files using the bamCoverage to visualize these data on the UCSC Genome Browser.^49^ Data (reads/peak) were normalized and differential analysis was carried out using the R package DESeq2.^50^ ATAC‐seq reads/peak were overlapped with DMPs and DMRs using bedtools.^42^

### Cytokine output and data analysis

Supernatants from nCD4 T‐cell cultures were thawed, and cytokines were quantitated using the Human Soluble Protein Flex Set Cytometric Bead Array (BD Biosciences) according to the manufacturer's instructions. Cytometric bead array data were measured on an LSR II X‐20 Fortessa and analyzed using the FCAP Array Software. A total of five cytokines (IL2, IL6, IL10, IFN‐γ, TNF‐α) were measured in both infants and adolescents using this approach.^6^, ^18^ To ensure that the cytokine data were comparable across the two datasets, and to minimize potential variation due to batch effects, we calculated the fold change in cytokine concentrations between quiescent and activated samples for each individual, and for each cytokine measured.

## AUTHOR CONTRIBUTIONS

SI wrote the main manuscript text, analyzed all of the data, prepared Figures 2, 3, 4, 5, 6 and generated DNA methylation data from adolescent samples. MN performed cell isolation and culture experiments, and DM generated DNA methylation data from infant samples. BN contributed to Figure 1, and provided analysis guidelines. All authors reviewed the manuscript.

## CONFLICT OF INTEREST

The authors declare no conflicts of interest.

## Supporting information

 

## Data Availability

The data assessed in this study are publicly available on NCBI's Gene Expression Omnibus (GEO) and are accessible through GEO Series accession number GSE114135 for the infant DNA methylation and transcriptomic data; and GSE189148 for the adolescent methylation data. Publicly available ATAC‐seq data from unrelated infants was downloaded from the GEO database as well, accession number GSE157174. Cytokine data from the infants and adolescents are supplied in the supplementary materials; along with details of differentially methylated probes and regions.
