# Supplementary material for: Epigenomic variability is associated with age‐specific naïve CD4 T cell response to activation in infants and adolescents
Source: Immunol Cell Biol. 2023 Mar 1;101(5):397–411. doi: 10.1111/imcb.12628 (PMC10952707; doi:10.1111/imcb.12628)
Supplement: Supplementary file 1 [file IMCB-101-397-s001.docx]

**Supplementary materials:** Immuno-epigenomic analysis identifies attenuated interferon responses in naïve CD4 T cells of adolescents with peanut and multi-food allergy

Samira Imran^1^, Melanie R Neeland^1^, Stephen Peng, Amanda Vlahos^1^, David Martino^1,2^, Shyamali C Dharmage^1,4^, Mimi LK Tang^1,3^, Susan Sawyer^1,5^, Thanh Dang^1^, Vicki McWilliam^1,3^, Rachel L Peters^1^, Jennifer J Koplin^1^, Kirsten P Perrett^1,3^, Boris Novakovic^1*^, Richard Saffery^1*^

**
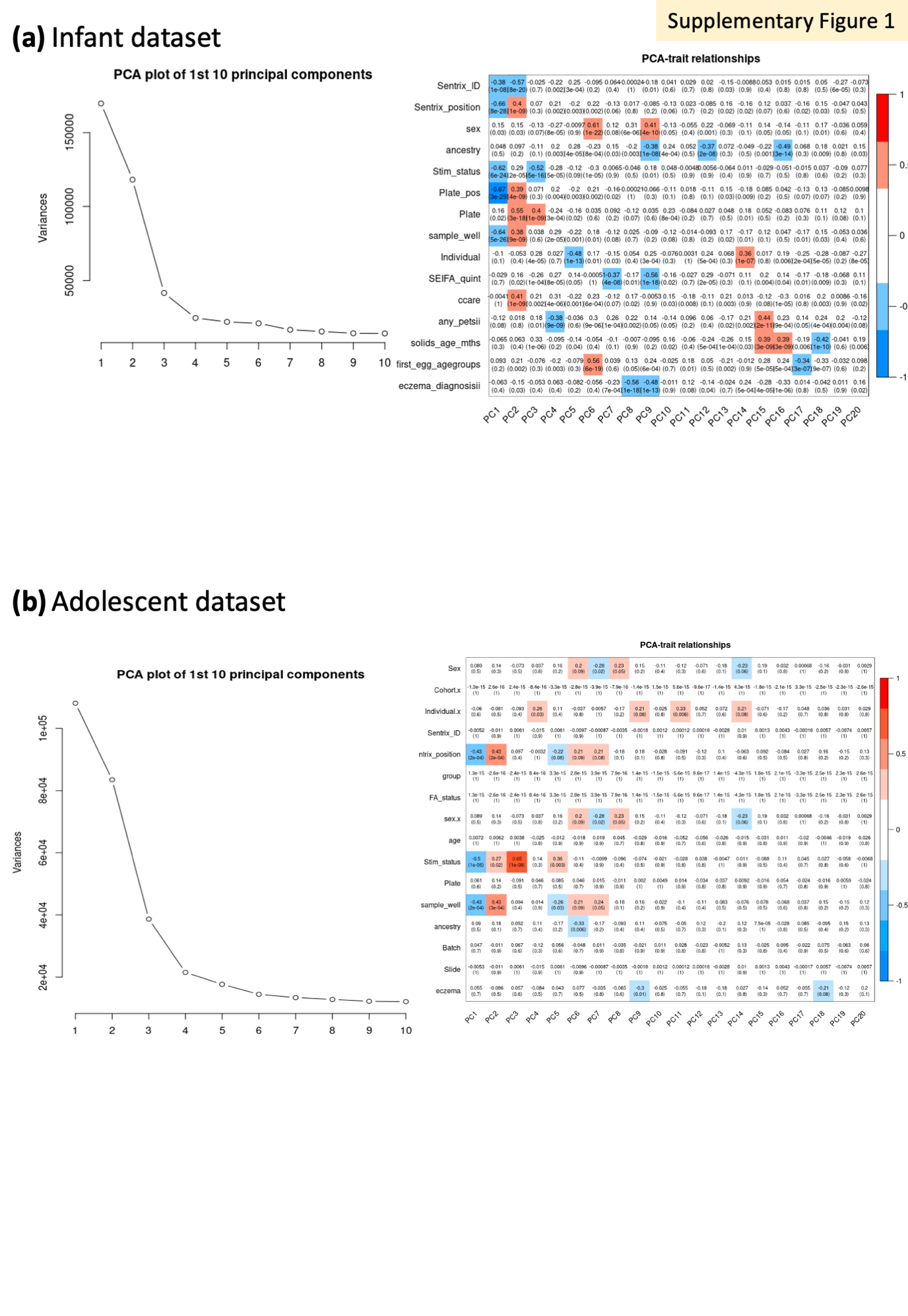
 Supplementary figure 1:** Plot of first 10 principal components and their contribution to variation of sample and heatmap of contribution of each trait to principal components in **(a)** Infant dataset and **(b)** Adolescent dataset

**
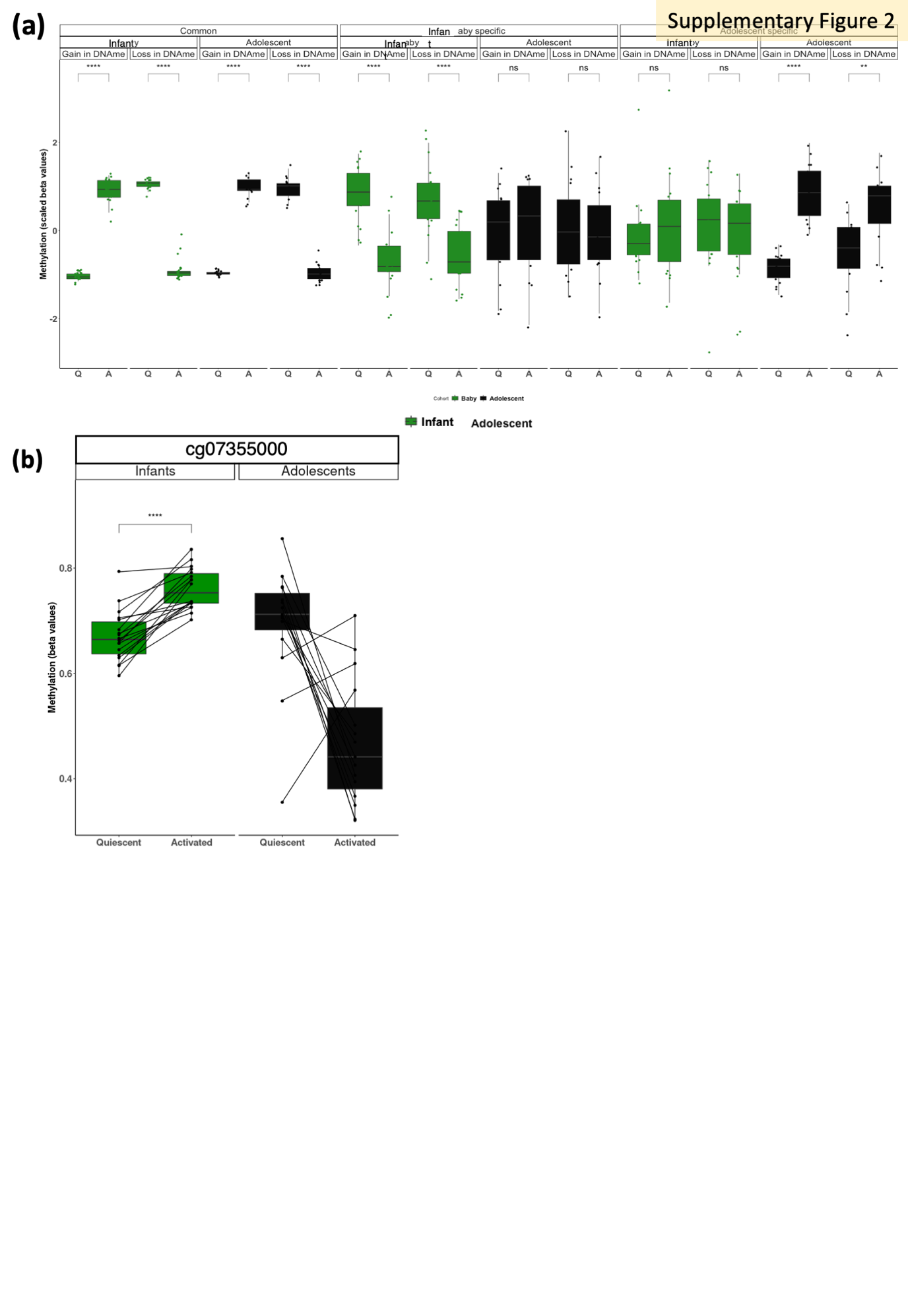
**

**Supplementary figure 2:** **(a)** Boxplots of methylation values of samples across probes showing shared, infant-specific and adolescent-specific patterns of activation, separated by age group, and into groups of probes showing loss or gain in methylation following activation. **(b)** Boxplot of methylation values of samples for probe (cg07355000) showing opposing trend in methylation in infants and adolescents, separated by age group. Lines connect paired samples.

**
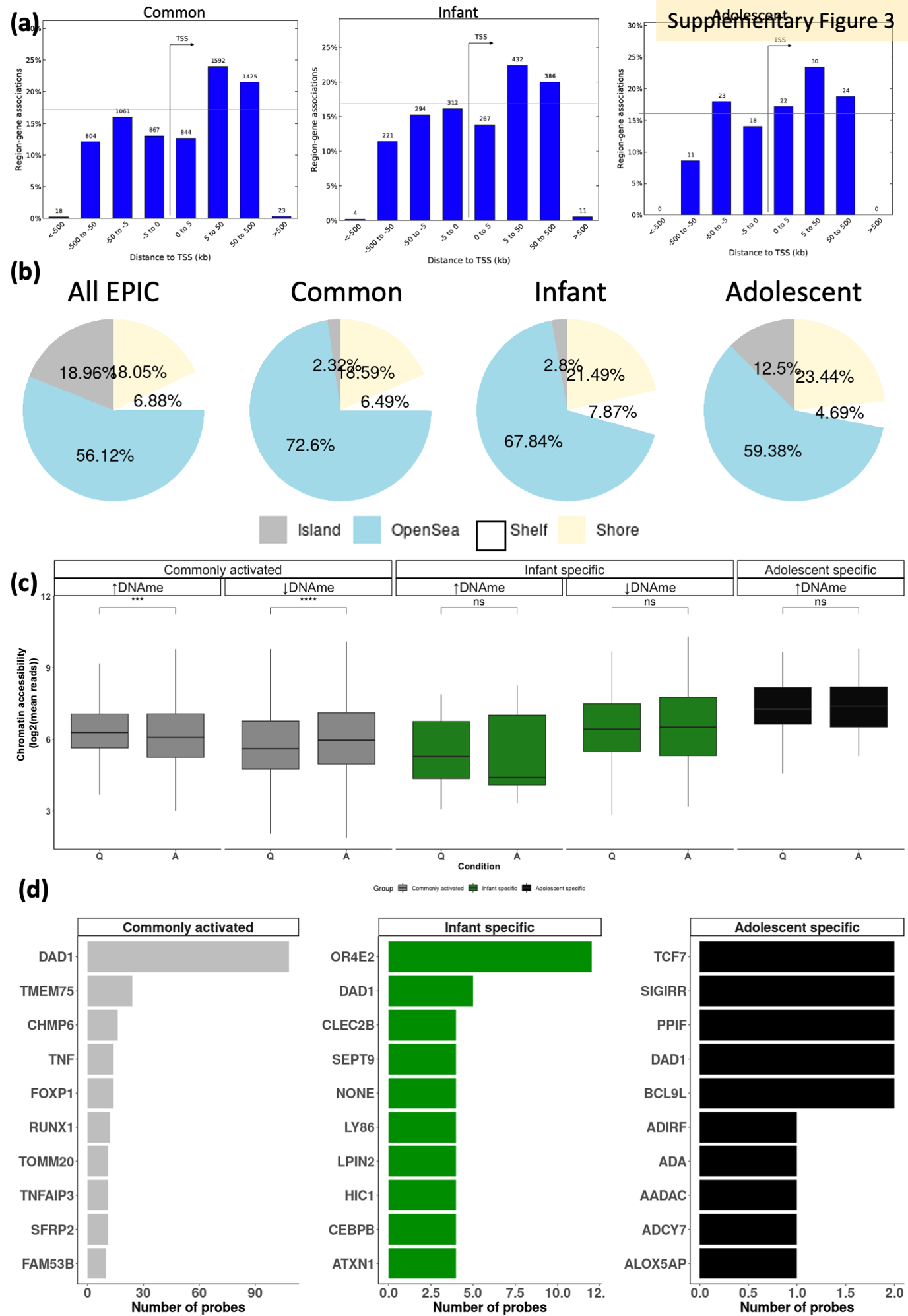
**

**Supplementary figure 3: (a)** Barplot of location of probes relative to gene transcription start sites. **(b)** Pie chart of distribution of sets of probes relative to CpG islands. **(c)** Boxplots of fold-change of ATAC-peak reads at probes showing shared, infant-specific, and adolescent specific responses separated into groups of probes showing loss of methylation following activation, and those showing a gain in methylation following activation. Values displayed above boxplots represent *P-*values determined by the Mann–Whitney *U*-test between groups.

**
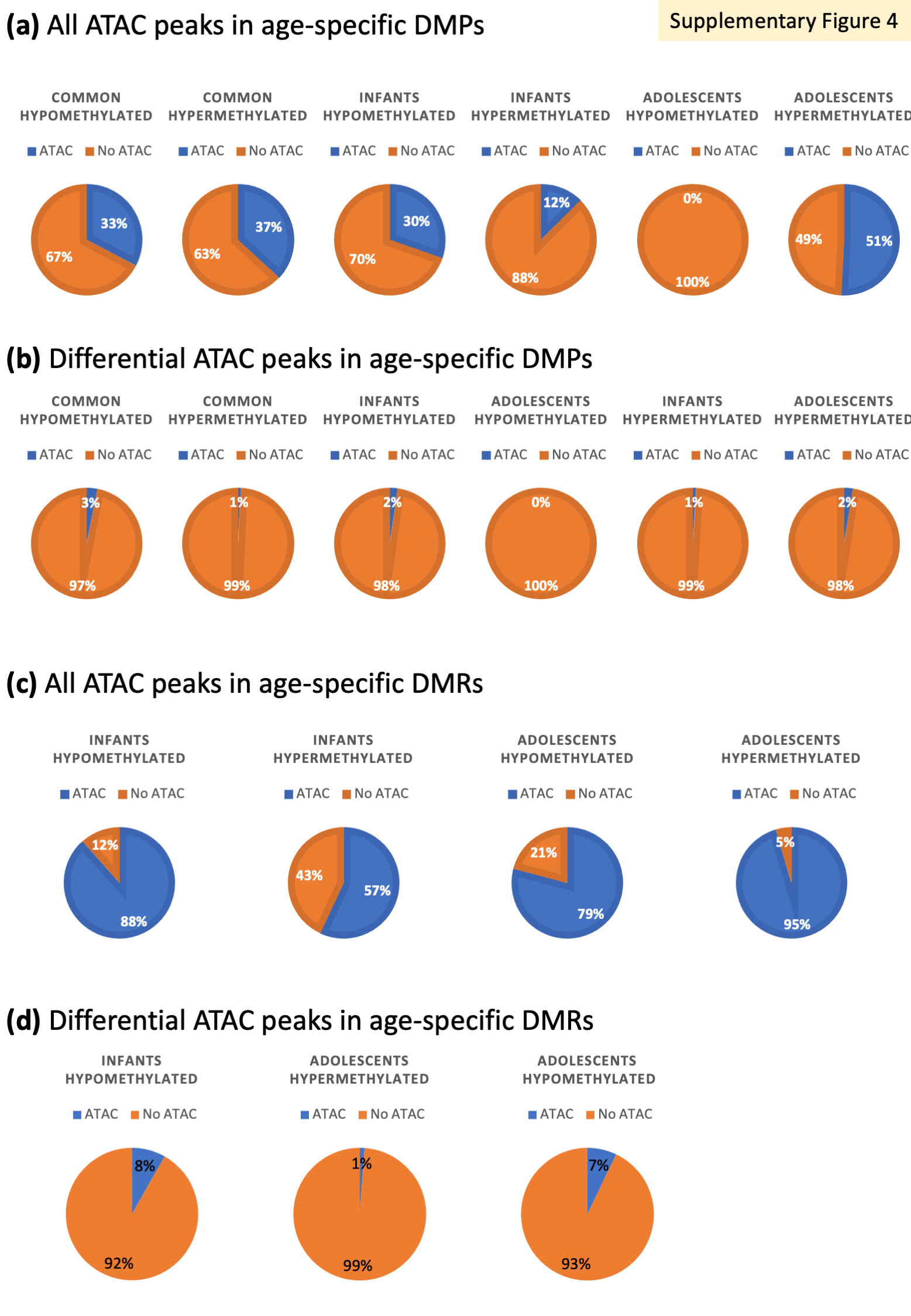
**

**Supplementary figure 4:** Distribution of **(a)** All ATAC peaks **(b)** Differential ATAC peaks across sets of DMPs. Distribution of **(c)** All ATAC peaks **(d)** Differential ATAC peaks across sets of DMRs.

**
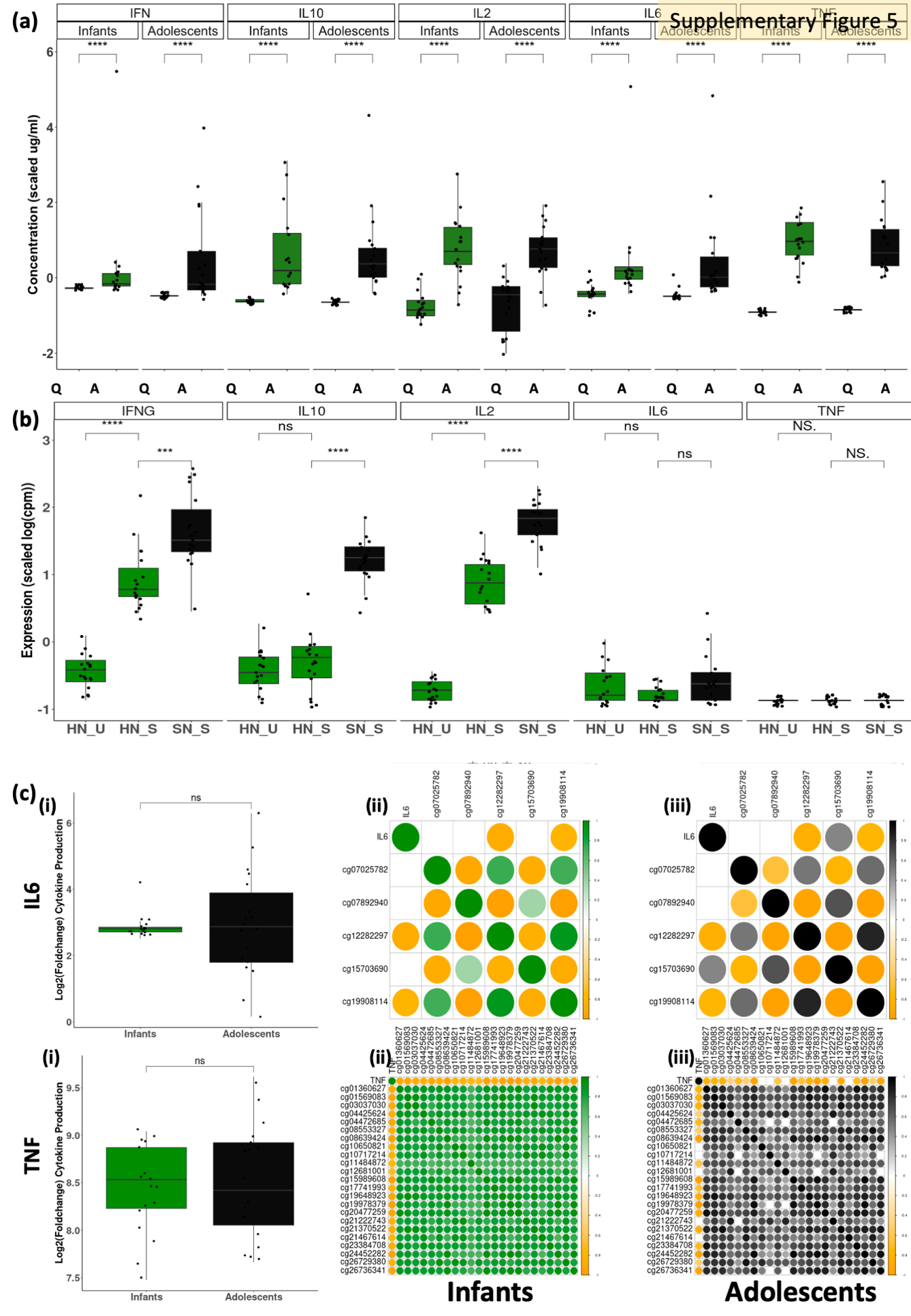
**

**Supplementary figure 5: (a)** Boxplot of scaled cytokine concentrations of infants and adolescents at quiescence and following activation. Values have been scaled to allow for comparability. **(b)** Gene expression (scaled logcpm) of selected cytokines infants and adolescents at quiescence and following activation. Values have been scaled to allow for comparability **(c)** **(i**) Boxplots of log2(fold-change) of cytokine production between quiescent and activated samples in infants and adolescents. **(ii)** Correlation plot of cytokine levels and methylation of age-specific DMPs near gene encoding cytokine in infant samples. **(iii)** Correlation plot of cytokine levels and methylation of age-specific DMPs near gene encoding cytokine in adolescent samples.

**Supplementary table 1: List of all activation-associated DMPs, annotated with those showing *P-val* significance and/or reaching methylation difference cutoff >5% between quiescent and activated samples in infants and/or adolescents. Information regarding ATAC-peak distribution (stable as well as dynamic peaks indicated) are also included.**

| Probe | CTRL_mean_unstim_adolescent | CTRL_mean_stim_adolescent | CTRL_unstim_vs_stim_dB_adolescent | CTRL_mean_unstim_infants | CTRL_mean_stim_infants | CTRL_unstim_vs_stim_dB_infants | Pval_sig_HN | Pval_sig_SN | adj.P.Val_adolescent | adj.P.Val_infants | Significance_in_age_groups | Ageing_signature |
| --- | --- | --- | --- | --- | --- | --- | --- | --- | --- | --- | --- | --- |
| cg00001793 | 0.69553054 | 0.76702032 | 0.07148978 | 0.78439747 | 0.8527234 | 0.06832593 | Pvalue significant and dB significant in infants | Not significant in adolescents | 0.05407225 | 6.05E-06 | Pvalue significant and dB significant in infants_Not significant in adolescents | Not Associated |
| cg00001801 | 0.48712311 | 0.53101698 | 0.04389387 | 0.54338662 | 0.60640516 | 0.06301854 | Pvalue significant and dB significant in infants | Not significant in adolescents | 0.77471455 | 0.00448424 | Pvalue significant and dB significant in infants_Not significant in adolescents | Not Associated |
| cg00001854 | 0.87617634 | 0.83647299 | -0.0397034 | 0.90179833 | 0.83924982 | -0.0625485 | Pvalue significant and dB significant in infants | Not significant in adolescents | 0.01629772 | 6.19E-08 | Pvalue significant and dB significant in infants_Not significant in adolescents | Not Associated |
| cg00002930 | 0.10771032 | 0.08704066 | -0.0206697 | 0.14739995 | 0.12577654 | -0.0216234 | Not significant in infants | Only P value significant in adolescents | 0.00914849 | 0.06023231 | Not significant in infants_Only P value significant in adolescents | Not Associated |
| cg00004667 | 0.43084591 | 0.11183922 | -0.3190067 | 0.63331951 | 0.16412704 | -0.4691925 | Pvalue significant and dB significant in infants | Pvalue significant and dB significant in adolescents | 6.17E-11 | 5.35E-16 | Pvalue and dB significant in both | Not Associated |
| cg00004723 | 0.84600435 | 0.82705298 | -0.0189514 | 0.88662922 | 0.81729008 | -0.0693391 | Pvalue significant and dB significant in infants | Not significant in adolescents | 0.1025769 | 5.62E-06 | Pvalue significant and dB significant in infants_Not significant in adolescents | Not Associated |
| cg00005597 | 0.24050782 | 0.22803724 | -0.0124706 | 0.29804854 | 0.22621709 | -0.0718315 | Pvalue significant and dB significant in infants | Not significant in adolescents | 0.48059685 | 0.00042032 | Pvalue significant and dB significant in infants_Not significant in adolescents | Not Associated |
| cg00005599 | 0.46984518 | 0.28290571 | -0.1869395 | 0.79509961 | 0.57308874 | -0.2220109 | Pvalue significant and dB significant in infants | Pvalue significant and dB significant in adolescents | 9.73E-05 | 2.65E-10 | Pvalue and dB significant in both | Not Associated |
| cg00007076 | 0.70275134 | 0.59453664 | -0.1082147 | 0.71215617 | 0.57271371 | -0.1394425 | Pvalue significant and dB significant in infants | Pvalue significant and dB significant in adolescents | 0.00011492 | 9.76E-11 | Pvalue and dB significant in both | Not Associated |
| cg00008665 | 0.8195768 | 0.78435489 | -0.0352219 | 0.87381954 | 0.81381826 | -0.0600013 | Pvalue significant and dB significant in infants | Not significant in adolescents | 0.19886518 | 3.40E-05 | Pvalue significant and dB significant in infants_Not significant in adolescents | Not Associated |
| cg00009349 | 0.64594336 | 0.57311731 | -0.072826 | 0.785649 | 0.68237957 | -0.1032694 | Pvalue significant and dB significant in infants | Not significant in adolescents | 0.10003743 | 1.69E-05 | Pvalue significant and dB significant in infants_Not significant in adolescents | Not Associated |
| cg00009602 | 0.50140318 | 0.4069336 | -0.0944696 | 0.77813638 | 0.55924672 | -0.2188897 | Pvalue significant and dB significant in infants | Not significant in adolescents | 0.02410902 | 7.41E-11 | Pvalue significant and dB significant in infants_Not significant in adolescents | Not Associated |
| cg00010187 | 0.07238347 | 0.12870292 | 0.05631945 | 0.09229752 | 0.15739266 | 0.06509514 | Pvalue significant and dB significant in infants | Pvalue significant and dB significant in adolescents | 0.00013161 | 1.38E-07 | Pvalue and dB significant in both | Not Associated |
| cg00012692 | 0.1814146 | 0.24288517 | 0.06147057 | 0.24988205 | 0.27462562 | 0.02474357 | Not significant in infants | Pvalue significant and dB significant in adolescents | 0.00065414 | 0.01722939 | Not significant in infants_Pvalue significant and dB significant in adolescents | Not Associated |
| cg00013899 | 0.91739681 | 0.86873724 | -0.0486596 | 0.92510172 | 0.87191922 | -0.0531825 | Pvalue significant and dB significant in infants | Only P value significant in adolescents | 0.00182452 | 9.24E-06 | Pvalue significant and dB significant in infants_Only P value significant in adolescents | Not Associated |
| cg00015664 | 0.80628853 | 0.68930555 | -0.116983 | 0.80912559 | 0.6692098 | -0.1399158 | Pvalue significant and dB significant in infants | Pvalue significant and dB significant in adolescents | 0.00065093 | 6.04E-06 | Pvalue and dB significant in both | Not Associated |
| cg00017639 | 0.75990735 | 0.75599882 | -0.0039085 | 0.83643008 | 0.78152147 | -0.0549086 | Pvalue significant and dB significant in infants | Not significant in adolescents | 0.70396558 | 0.00313648 | Pvalue significant and dB significant in infants_Not significant in adolescents | Not Associated |
| cg00019275 | 0.81773218 | 0.79252103 | -0.0252111 | 0.85028034 | 0.79525395 | -0.0550264 | Pvalue significant and dB significant in infants | Not significant in adolescents | 0.55810707 | 0.00567227 | Pvalue significant and dB significant in infants_Not significant in adolescents | Not Associated |
| cg00020991 | 0.82232277 | 0.78409963 | -0.0382231 | 0.89959906 | 0.84302077 | -0.0565783 | Pvalue significant and dB significant in infants | Not significant in adolescents | 0.15819997 | 4.83E-06 | Pvalue significant and dB significant in infants_Not significant in adolescents | Not Associated |
| cg00023464 | 0.64333617 | 0.60038453 | -0.0429516 | 0.70357537 | 0.6459798 | -0.0575956 | Pvalue significant and dB significant in infants | Not significant in adolescents | 0.27706823 | 0.00160707 | Pvalue significant and dB significant in infants_Not significant in adolescents | Not Associated |
| cg00024674 | 0.81648343 | 0.77613479 | -0.0403486 | 0.88790854 | 0.82862669 | -0.0592818 | Pvalue significant and dB significant in infants | Not significant in adolescents | 0.05692925 | 3.81E-07 | Pvalue significant and dB significant in infants_Not significant in adolescents | Not Associated |
| cg00024937 | 0.78638099 | 0.77263348 | -0.0137475 | 0.86607863 | 0.81514346 | -0.0509352 | Pvalue significant and dB significant in infants | Not significant in adolescents | 0.68189851 | 0.00099491 | Pvalue significant and dB significant in infants_Not significant in adolescents | Not Associated |
| cg00029521 | 0.83197044 | 0.81096533 | -0.0210051 | 0.88830593 | 0.83675026 | -0.0515557 | Pvalue significant and dB significant in infants | Not significant in adolescents | 0.30460725 | 6.23E-06 | Pvalue significant and dB significant in infants_Not significant in adolescents | Not Associated |
| cg00030290 | 0.469666 | 0.37100882 | -0.0986572 | 0.54131744 | 0.42554496 | -0.1157725 | Pvalue significant and dB significant in infants | Pvalue significant and dB significant in adolescents | 0.0008086 | 4.21E-07 | Pvalue and dB significant in both | Not Associated |
| cg00032366 | 0.7559514 | 0.70374114 | -0.0522103 | 0.81497851 | 0.74723365 | -0.0677449 | Pvalue significant and dB significant in infants | Not significant in adolescents | 0.09766299 | 1.31E-05 | Pvalue significant and dB significant in infants_Not significant in adolescents | Not Associated |
| cg00032609 | 0.37879313 | 0.34956204 | -0.0292311 | 0.42848624 | 0.33771551 | -0.0907707 | Pvalue significant and dB significant in infants | Not significant in adolescents | 0.4164348 | 6.76E-06 | Pvalue significant and dB significant in infants_Not significant in adolescents | Not Associated |
| cg00032703 | 0.71582286 | 0.66972194 | -0.0461009 | 0.70721141 | 0.62374663 | -0.0834648 | Pvalue significant and dB significant in infants | Not significant in adolescents | 0.14198571 | 2.43E-05 | Pvalue significant and dB significant in infants_Not significant in adolescents | Not Associated |
| cg00033818 | 0.75558583 | 0.54539897 | -0.2101869 | 0.71752446 | 0.63847973 | -0.0790447 | Pvalue significant and dB significant in infants | Pvalue significant and dB significant in adolescents | 1.59E-05 | 0.00246939 | Pvalue and dB significant in both | Not Associated |
| cg00033992 | 0.56204527 | 0.57826538 | 0.01622012 | 0.55966743 | 0.63047385 | 0.07080642 | Pvalue significant and dB significant in infants | Not significant in adolescents | 0.79788782 | 1.81E-05 | Pvalue significant and dB significant in infants_Not significant in adolescents | Not Associated |
| cg00037187 | 0.85148427 | 0.80049946 | -0.0509848 | 0.8875325 | 0.85431613 | -0.0332164 | Only P value significant in infants | Pvalue significant and dB significant in adolescents | 0.00076893 | 0.00049513 | Only P value significant in infants_Pvalue significant and dB significant in adolescents | Not Associated |
| cg00038791 | 0.52340877 | 0.58305544 | 0.05964667 | 0.49129982 | 0.60233956 | 0.11103974 | Pvalue significant and dB significant in infants | Not significant in adolescents | 0.15946783 | 1.65E-07 | Pvalue significant and dB significant in infants_Not significant in adolescents | Not Associated |
| cg00041030 | 0.48848218 | 0.2099052 | -0.278577 | 0.56080204 | 0.18453459 | -0.3762675 | Pvalue significant and dB significant in infants | Pvalue significant and dB significant in adolescents | 1.07E-10 | 2.33E-14 | Pvalue and dB significant in both | Not Associated |
| cg00041829 | 0.59859367 | 0.5750128 | -0.0235809 | 0.8097727 | 0.75445225 | -0.0553205 | Pvalue significant and dB significant in infants | Not significant in adolescents | 0.47536897 | 0.00546647 | Pvalue significant and dB significant in infants_Not significant in adolescents | Not Associated |

**Abbreviated table, please contact corresponding author for full table.

**Supplementary table 2:** Results of functional enrichment analysis (in GO and KEGG databases) of genes linked to probes showing shared and age-specific patterns of activation

| Enriched KEGG terms among genes associated with commonly activated probes | |  | |  | | |  | |  | |
| --- | --- | --- | --- | --- | --- | --- | --- | --- | --- | --- |
|  | Description | | N | | DE | P.DE | | FDR | | SigGenesInSet |
| path:hsa04660 | T cell receptor signaling pathway | | 102 | | 45 | 3.20E-09 | | 1.10E-06 | | VAV3,MALT1,MAP3K8,CSF2,CTLA4,DLG1,FYN,LAT,GRB2,GSK3B,IL2,IL5,IL10,ITK,RHOA,LCP2,NFATC1,NFATC2,NFKB1,NFKBIA,NFKBIE,PAK1,PDCD1,PIK3CA,PIK3CD,PIK3R1,PLCG1,PPP3CA,PRKCQ,MAPK1,MAP2K2,PAK6,PTPN6,TNF,ZAP70,NCK2,CARD11,PIK3R3,CBLB,MAP3K14,CD3E,CD247,CD4,CD28,GRAP2 |
| path:hsa04659 | Th17 cell differentiation | | 106 | | 42 | 1.07E-07 | | 1.83E-05 | | IL23R,AHR,MTOR,GATA3,LAT,TBX21,HIF1A,HSP90AA1,IFNGR2,IL1R1,IL1RAP,IL2,IL2RA,IL2RB,IL4R,IL6R,IRF4,JAK1,SMAD2,SMAD3,SMAD4,NFATC1,NFATC2,NFKB1,NFKBIA,NFKBIE,IL21R,PLCG1,PPP3CA,PRKCQ,MAPK1,RARA,RORA,RXRA,STAT3,STAT5B,TGFB1,ZAP70,RUNX1,CD3E,CD247,CD4 |
| path:hsa05131 | Shigellosis | | 244 | | 70 | 7.33E-07 | | 7.84E-05 | | ACTR3,WASF2,CALCOCO2,TNIP1,TAB1,SEPTIN9,MALT1,NLRP3,CSF2,DIAPH1,DOCK1,U2AF1L4,ATG14,FNBP1,FOXO1,FOXO3,TAB2,PLCB1,FBXW11,MTOR,CYTH4,GSK3B,HK2,HK3,IL1R1,IL18,ITGA5,ITPR1,ITPR2,RHOA,ATM,NFKB1,NFKBIA,PFN1,PIK3CA,PIK3CD,PIK3R1,PLCG1,RNF31,SEPTIN11,PRKCE,PRKCQ,MAPK1,PTK2,RPTOR,PXN,RRAGD,BCL2,BCL2L1,CCL5,SKP1,TNF,TP53,TRAF2,TRAF5,UBC,UBE2N,UBE2V1,CAPN1,CAPN2,TLN2,PIK3R3,ACTN1,RIPK2,SQSTM1,BTRC,RPS6KA5,CYTH3,CYTH1,CD44,ELMO1 |
| path:hsa04621 | NOD-like receptor signaling pathway | | 182 | | 49 | 9.14E-07 | | 7.84E-05 | | TAB1,NLRP3,ANTXR2,NEK7,CTSB,NLRP1,TAB2,PLCB1,GABARAPL1,PANX1,CXCL3,BIRC2,BIRC3,HSP90AA1,IFI16,IL18,IRF7,ITPR1,ITPR2,JAK1,RHOA,NFKB1,NFKBIA,OAS1,OAS2,TRPV2,RNF31,PKN2,ERBIN,MAPK1,BCL2,BCL2L1,CCL5,NOD2,TNF,TNFAIP3,TRAF2,TRAF3,TRAF5,TRPM2,YWHAE,NLRX1,CASP8,ANTXR1,RIPK2,PSTPIP1,MCU,AIM2,IKBKE |
| path:hsa05135 | Yersinia infection | | 135 | | 47 | 3.76E-06 | | 0.000258 | | ACTR3,WASF2,VAV3,TAB1,NLRP3,DOCK1,PTK2B,TAB2,ARHGEF12,FYB1,LAT,GNAQ,GSK3B,IL2,IL10,IL18,ITGA5,RHOA,RHOG,LCP2,LIMK1,NFATC1,NFATC2,NFKB1,NFKBIA,PIK3CA,PIK3CD,PIK3R1,PLCG1,PKN2,MAPK1,MAP2K2,MAP2K3,PTK2,PXN,RAC2,MAP2K4,TNF,TRAF2,WIPF1,ZAP70,PIP5K1A,PIK3R3,ARHGEF7,CD4,GIT2,ELMO1 |
| path:hsa04060 | Cytokine-cytokine receptor interaction | | 290 | | 56 | 5.00E-06 | | 0.00028609 | | CCR9,EDAR,IL24,CCR1,CCR5,CCR7,CCR8,IL31RA,CSF2,CTF1,IL23R,IL17RA,AMHR2,TNFRSF21,CXCL17,CXCL3,IL19,IFNGR2,IL1R1,IL1RAP,IL1RN,IL2,IL2RA,IL2RB,IL4R,IL5,IL6R,IL10,IL12RB2,IL16,TNFRSF9,IL18,CXCL10,IL21R,IL17D,IL36G,CCL5,CXCR5,TGFB1,TNF,TNFRSF1B,TNFSF4,TNFRSF4,IL1R2,CXCR4,RELT,TNFSF14,TNFRSF10B,IL18RAP,IL1RL2,ACVR1,ACVR1B,CD4,IL32,CD27,TNFRSF8,TNFSF8 |
| path:hsa04064 | NF-kappa B signaling pathway | | 103 | | 33 | 2.38E-05 | | 0.00111574 | | TAB1,MALT1,EDAR,TIRAP,ERC1,TAB2,LAT,CXCL3,BIRC2,BIRC3,TICAM2,IL1R1,LYN,GADD45B,ATM,NFKB1,NFKBIA,PLCG1,PRKCB,PRKCQ,BCL2,BCL2A1,BCL2L1,TNF,TNFAIP3,TRAF1,TRAF2,TRAF3,TRAF5,ZAP70,CARD11,TNFSF14,CFLAR,MAP3K14 |
| path:hsa05235 | PD-L1 expression and PD-1 checkpoint pathway in cancer | | 89 | | 32 | 2.60E-05 | | 0.00111574 | | BATF,TIRAP,MTOR,LAT,CD274,HIF1A,IFNGR2,TICAM2,JAK1,NFATC1,NFATC2,NFKB1,NFKBIA,NFKBIE,PDCD1,PIK3CA,PIK3CD,PIK3R1,PLCG1,PPP3CA,PRKCQ,MAPK1,MAP2K2,MAP2K3,PTEN,PTPN6,STAT3,ZAP70,PIK3R3,CD3E,CD247,CD4,CD28 |
| path:hsa05417 | Lipid and atherosclerosis | | 215 | | 56 | 3.97E-05 | | 0.00151304 | | TLR6,VAV3,TAB1,NLRP3,TIRAP,ERN1,ATF6,TAB2,PLCB1,CXCL3,GSK3B,ERO1A,HSPA8,HSP90AA1,TICAM2,IL18,IRF7,ITPR1,RHOA,LDLR,LYN,MAP3K5,NCF4,NFATC1,NFATC2,NFKB1,NFKBIA,NOS3,PIK3CA,PIK3CD,PIK3R1,PLCG1,POU2F1,POU2F2,PPP3CA,PRKCA,MAPK1,MAP2K3,PTK2,BCL2,BCL2L1,RXRA,CCL5,MAP2K4,SOD2,STAT3,TNF,TP53,TRAF2,TRAF3,CAMK2D,CASP6,CASP7,CASP8,PIK3R3,TNFRSF10B,ABCG1,IKBKE |
| path:hsa04062 | Chemokine signaling pathway | | 192 | | 53 | 4.54E-05 | | 0.00155889 | | RASGRP2,VAV3,GNB5,CCR9,CCR1,CCR5,CCR7,CCR8,PIK3R6,GRK3,PTK2B,FGR,FOXO3,PLCB1,PIK3R5,GNAQ,GNG7,GNGT2,GRK5,GRK6,GRB2,CXCL3,GSK3B,CXCL10,ITK,RHOA,SHC4,LYN,ARRB1,NFKB1,NFKBIA,PAK1,PIK3CA,PIK3CD,PIK3CG,PIK3R1,PLCG1,SHC3,GNG2,PRKCB,MAPK1,GNG12,PARD3,PTK2,PXN,RAC2,CCL5,CXCR5,STAT3,STAT5B,TIAM1,CXCR4,PIK3R3,ELMO1 |
| path:hsa04061 | Viral protein interaction with cytokine and cytokine receptor | | 99 | | 22 | 5.44E-05 | | 0.00169587 | | CCR9,IL24,CCR1,CCR5,CCR7,CCR8,CXCL3,IL19,IL2,IL2RA,IL2RB,IL6R,IL10,IL18,CXCL10,CCL5,CXCR5,TNF,TNFRSF1B,CXCR4,TNFSF14,TNFRSF10B,IL18RAP |
| path:hsa04210 | Apoptosis | | 135 | | 40 | 7.04E-05 | | 0.00201249 | | BCL2L11,PARP3,CTSB,CTSZ,ERN1,BBC3,BIRC2,BIRC3,ITPR1,ITPR2,LMNA,MAP3K5,GADD45B,ATM,NFKB1,NFKBIA,NTRK1,PIK3CA,PIK3CD,PIK3R1,MAPK1,MAP2K2,DIABLO,BCL2,BCL2A1,BCL2L1,TNF,TP53,TRAF1,TRAF2,CAPN1,CAPN2,CASP6,CASP7,CASP8,TUBA1C,LMNB2,PIK3R3,TNFRSF10B,CFLAR,MAP3K14 |
| path:hsa04650 | Natural killer cell mediated cytotoxicity | | 124 | | 35 | 8.62E-05 | | 0.00207138 | | VAV3,CSF2,PTK2B,FYN,LAT,GRB2,HLA-E,IFNGR2,ITGAL,ITGB2,LCP2,SHC4,NFATC1,NFATC2,PAK1,CD244,PIK3CA,PIK3CD,PIK3R1,PLCG1,SHC3,PPP3CA,PRKCA,PRKCB,MAPK1,MAP2K2,PTPN6,RAC2,TNF,TYROBP,ZAP70,PIK3R3,TNFRSF10B,CD247,NCR2 |
| path:hsa05321 | Inflammatory bowel disease | | 63 | | 22 | 9.38E-05 | | 0.00207138 | | IL23R,GATA3,TBX21,IFNGR2,IL2,IL4R,IL5,IL10,IL12RB2,IL18,SMAD2,SMAD3,NFATC1,NFKB1,IL21R,RORA,NOD2,STAT3,STAT4,TGFB1,TNF,IL18RAP |
| path:hsa05203 | Viral carcinogenesis | | 193 | | 53 | 9.59E-05 | | 0.00207138 | | CDK6,CHD4,CCR5,CCR8,CREBBP,ATF6B,DLG1,EGR3,UBR4,SND1,GRB2,GTF2E2,GTF2H4,H2BC5,HLA-E,HPN,IRF7,JAK1,RHOA,LYN,NFKB1,NFKBIA,HDAC7,PIK3CA,PIK3CD,PIK3R1,PKM,VAC14,MAPK1,PXN,RASA2,RBL1,REL,CREB3L2,STAT3,STAT5B,TP53,TRAF1,TRAF2,TRAF3,TRAF5,YWHAE,HDAC11,MAD1L1,CASP8,PIK3R3,ACTN1,KAT2B,CCND2,CCND3,ATP6V0D1,MAPKAPK2,TBPL1,HDAC4 |
| path:hsa05170 | Human immunodeficiency virus 1 infection | | 211 | | 56 | 9.66E-05 | | 0.00207138 | | TAB1,GNB5,CCR5,PTK2B,TAB2,FBXW11,MTOR,SAMHD1,GNAQ,GNG7,GNGT2,PDIA3,HLA-E,ITPR1,ITPR2,LIMK1,LIMK2,ATM,NFATC1,NFATC2,NFATC4,NFKB1,NFKBIA,PAK1,PIK3CA,PIK3CD,PIK3R1,PLCG1,GNG2,ATR,PPP3CA,PRKCA,PRKCB,MAPK1,GNG12,MAP2K2,MAP2K3,PAK6,PTK2,PXN,RAC2,BCL2,BCL2L1,SKP1,TAP1,TAP2,TNF,TNFRSF1B,TRAF2,TRAF5,CXCR4,CASP8,PIK3R3,BTRC,CD3E,CD247,CD4 |
| path:hsa04668 | TNF signaling pathway | | 112 | | 34 | 0.000111712 | | 0.00225396 | | TAB1,MAP3K8,ATF6B,CSF2,TAB2,CXCL3,BIRC2,BIRC3,CXCL10,MAP3K5,NFKB1,NFKBIA,PIK3CA,PIK3CD,PIK3R1,MAPK1,MAP2K3,CCL5,NOD2,MAP2K4,CREB3L2,TNF,TNFAIP3,TNFRSF1B,TRAF1,TRAF2,TRAF3,TRAF5,ITCH,CASP7,CASP8,PIK3R3,CFLAR,MAP3K14,RPS6KA5 |
| path:hsa05167 | Kaposi sarcoma-associated herpesvirus infection | | 194 | | 52 | 0.000169876 | | 0.00323709 | | CDK6,GNB5,CCR1,CCR5,CCR8,CREBBP,CSF2,PIK3R6,RCAN1,E2F2,ATG14,PIK3R5,MTOR,GNG7,GNGT2,CXCL3,GSK3B,HIF1A,HLA-E,IRF7,ITPR1,ITPR2,JAK1,LYN,NFATC1,NFATC2,NFATC4,NFKB1,NFKBIA,LEF1,PIK3CA,PIK3CD,PIK3CG,PIK3R1,PLCG1,GNG2,PPP3CA,MAPK1,GNG12,MAP2K2,MAP2K4,STAT3,TCF7,TCF7L2,TP53,TRAF2,TRAF3,UBC,ZFP36,CASP8,PIK3R3,MAPKAPK2,IKBKE |
| path:hsa04662 | B cell receptor signaling pathway | | 80 | | 27 | 0.000305748 | | 0.00551956 | | VAV3,MALT1,PIK3AP1,DAPP1,GRB2,GSK3B,INPP5D,LYN,NFATC1,NFATC2,NFKB1,NFKBIA,NFKBIE,PIK3CA,PIK3CD,PIK3R1,PPP3CA,PRKCB,MAPK1,MAP2K2,PTPN6,RAC2,CARD11,PIK3R3,IFITM1,CD72,CD79A |
| path:hsa05221 | Acute myeloid leukemia | | 67 | | 25 | 0.000365627 | | 0.00608791 | | CEBPE,CSF2,DUSP6,FLT3,MTOR,GRB2,ITGAM,JUP,NFKB1,LEF1,PIK3CA,PIM1,PIK3CD,PIK3R1,PML,MAPK1,MAP2K2,RARA,BCL2A1,STAT3,STAT5B,TCF7,TCF7L2,ZBTB16,PIK3R3,RUNX1 |
| Enriched KEGG terms among genes associated with infant-specific probes | |  | |  | | |  | |  | |
|  | Description | | N | | DE | P.DE | | FDR | | SigGenesInSet |
| path:hsa04071 | Sphingolipid signaling pathway | | 118 | | 20 | 0.002042278 | | 0.34764725 | | AKT3,SPTLC1,CTSD,S1PR1,MAP3K5,NFKB1,PIK3CD,PLD1,PPP2R2A,PPP2R3A,PPP2R5C,PRKCB,PRKCE,MAPK8,RAC2,BCL2,SGPP1,PIK3R3,ROCK2,GAB2 |
| path:hsa04668 | TNF signaling pathway | | 112 | | 17 | 0.002279591 | | 0.34764725 | | AKT3,MAP3K8,ATF2,CXCL10,IRF1,MAP3K5,NFKB1,NFKBIA,PIK3CD,MAPK8,MAP2K3,BCL3,CCL20,TRAF1,PIK3R3,CFLAR,MAP3K14,CREB5 |
| path:hsa04062 | Chemokine signaling pathway | | 192 | | 25 | 0.003202311 | | 0.34764725 | | AKT3,VAV3,CXCR6,ADCY5,ADCY7,ADCY9,CCR7,CRKL,GRK2,GRK5,GRK6,CXCL10,ITK,NFKB1,NFKBIA,PIK3CD,PRKACB,PRKCB,RAC2,CCL15,CCL20,SHC1,STAT3,STAT5B,PIK3R3,ROCK2 |
| path:hsa04928 | Parathyroid hormone synthesis, secretion and action | | 106 | | 19 | 0.004030693 | | 0.34764725 | | ADCY5,AKAP13,ADCY7,ADCY9,ATF2,HBEGF,GATA3,ITPR1,ITPR3,NR4A2,PDE4B,PLD1,PRKACB,PRKCB,BCL2,RXRA,RXRG,CREB5,ARHGEF11 |
| path:hsa04914 | Progesterone-mediated oocyte maturation | | 97 | | 15 | 0.006635893 | | 0.45787664 | | AKT3,ANAPC10,ADCY5,ADCY7,ADCY9,HSP90AB1,KIF22,PDE3B,PIK3CD,PRKACB,MAPK8,RPS6KA2,STK10,CPEB4,MAD1L1,PIK3R3 |
| path:hsa04659 | Th17 cell differentiation | | 106 | | 16 | 0.009054802 | | 0.46234559 | | GATA3,LAT,TBX21,HSP90AB1,IL2RB,IL6R,IL6ST,SMAD3,NFKB1,NFKBIA,MAPK8,RXRA,RXRG,STAT3,STAT5B,ZAP70 |
| path:hsa04064 | NF-kappa B signaling pathway | | 103 | | 14 | 0.010818001 | | 0.46234559 | | EDARADD,LAT,LTB,GADD45B,NFKB1,NFKBIA,PRKCB,BCL2,BCL2A1,TRAF1,UBE2I,ZAP70,CFLAR,MAP3K14 |
| path:hsa04935 | Growth hormone synthesis, secretion and action | | 119 | | 19 | 0.010824402 | | 0.46234559 | | AKT3,ADCY5,ADCY7,ADCY9,ATF2,CREBBP,CRKL,ITPR1,ITPR3,PIK3CD,PRKACB,PRKCB,MAPK8,MAP2K3,SHC1,STAT3,STAT5B,PIK3R3,CREB5 |
| path:hsa04210 | Apoptosis | | 135 | | 17 | 0.016248794 | | 0.46234559 | | AKT3,BCL2L11,CTSD,ITPR1,ITPR3,MAP3K5,GADD45B,NFKB1,NFKBIA,PIK3CD,SEPTIN4,MAPK8,BCL2,BCL2A1,TRAF1,PIK3R3,CFLAR,MAP3K14 |
| path:hsa04213 | Longevity regulating pathway - multiple species | | 62 | | 11 | 0.017426955 | | 0.46234559 | | AKT3,ADCY5,ADCY7,ADCY9,EIF4EBP2,PRKAG2,PIK3CD,PRKACB,RPTOR,CLPB,PIK3R3 |
| path:hsa05223 | Non-small cell lung cancer | | 72 | | 13 | 0.017902071 | | 0.46234559 | | AKT3,CDK6,EML4,GADD45B,PIK3CD,PRKCB,RARB,RXRA,RXRG,STAT3,STAT5B,RASSF5,PIK3R3 |
| path:hsa04666 | Fc gamma R-mediated phagocytosis | | 96 | | 15 | 0.017997785 | | 0.46234559 | | AKT3,ACTR3,VAV3,CFL2,CRKL,LAT,MYO10,PIK3CD,PLD1,PRKCB,PRKCE,PTPRC,RAC2,PIK3R3,GAB2 |
| path:hsa04660 | T cell receptor signaling pathway | | 102 | | 15 | 0.022288976 | | 0.46234559 | | AKT3,VAV3,MAP3K8,LAT,ITK,LCP2,NFKB1,NFKBIA,PIK3CD,MAPK8,PTPRC,ZAP70,NCK2,PIK3R3,MAP3K14 |
| path:hsa04664 | Fc epsilon RI signaling pathway | | 67 | | 11 | 0.022341991 | | 0.46234559 | | AKT3,VAV3,ALOX5AP,LAT,LCP2,PIK3CD,MAPK8,MAP2K3,RAC2,PIK3R3,GAB2 |
| path:hsa00533 | Glycosaminoglycan biosynthesis - keratan sulfate | | 14 | | 4 | 0.022976667 | | 0.46234559 | | FUT8,B4GALT1,ST3GAL1,ST3GAL3 |
| path:hsa04211 | Longevity regulating pathway | | 89 | | 14 | 0.02419445 | | 0.46234559 | | AKT3,ADCY5,ADCY7,ADCY9,ATF2,EIF4E,SESN1,NFKB1,PRKAG2,PIK3CD,PRKACB,RPTOR,TSC2,PIK3R3,CREB5 |
| path:hsa05222 | Small cell lung cancer | | 92 | | 14 | 0.026001974 | | 0.46234559 | | AKT3,CDK6,COL4A2,ITGB1,GADD45B,NFKB1,NFKBIA,PIK3CD,RARB,BCL2,RXRA,RXRG,TRAF1,PIK3R3 |
| path:hsa04931 | Insulin resistance | | 108 | | 15 | 0.026268171 | | 0.46234559 | | AKT3,NFKB1,NFKBIA,PRKAG2,PIK3CD,PTPA,PRKCB,PRKCE,MAPK8,PTPN1,PYGM,RPS6KA2,STAT3,PIK3R3,CREB5 |
| path:hsa04066 | HIF-1 signaling pathway | | 109 | | 15 | 0.026327782 | | 0.46234559 | | AKT3,EGLN2,CREBBP,EIF4E,ENO2,FLT1,GAPDH,HK1,IL6R,NFKB1,PIK3CD,PRKCB,BCL2,STAT3,ELOC,PIK3R3 |
| path:hsa04622 | RIG-I-like receptor signaling pathway | | 70 | | 8 | 0.027476927 | | 0.46234559 | | TANK,STING1,CXCL10,NFKB1,NFKBIA,MAPK8,NLRX1,ATG12,ISG15 |
| Enriched KEGG terms among genes associated with adolescent-specific probes | |  | |  | | |  | |  | |
|  | Description | | N | | DE | P.DE | | FDR | | SigGenesInSet |
| path:hsa04650 | Natural killer cell mediated cytotoxicity | | 124 | | 4 | 0.004505487 | | 0.57574783 | | ITGB2,KLRD1,PRKCB,CD247 |
| path:hsa04964 | Proximal tubule bicarbonate reclamation | | 23 | | 2 | 0.005834811 | | 0.57574783 | | GLS,PCK1 |
| path:hsa04070 | Phosphatidylinositol signaling system | | 97 | | 4 | 0.006621723 | | 0.57574783 | | INPP5A,ITPR1,PRKCB,DGKZ |
| path:hsa04015 | Rap1 signaling pathway | | 210 | | 6 | 0.006675337 | | 0.57574783 | | RASGRP2,ADCY7,ITGB2,PRKCB,SIPA1,SKAP1 |
| path:hsa04928 | Parathyroid hormone synthesis, secretion and action | | 106 | | 4 | 0.012573912 | | 0.73953885 | | ADCY7,ITPR1,LRP6,PRKCB |
| path:hsa04918 | Thyroid hormone synthesis | | 75 | | 3 | 0.016525484 | | 0.73953885 | | ADCY7,ITPR1,PRKCB |
| path:hsa04724 | Glutamatergic synapse | | 114 | | 4 | 0.019898684 | | 0.73953885 | | ADCY7,GLS,ITPR1,PRKCB |
| path:hsa04970 | Salivary secretion | | 93 | | 3 | 0.019954255 | | 0.73953885 | | ADCY7,ITPR1,PRKCB |
| path:hsa04972 | Pancreatic secretion | | 101 | | 3 | 0.022646416 | | 0.73953885 | | ADCY7,ITPR1,PRKCB |
| path:hsa04971 | Gastric acid secretion | | 76 | | 3 | 0.024414614 | | 0.73953885 | | ADCY7,ITPR1,PRKCB |
| path:hsa00470 | D-Amino acid metabolism | | 6 | | 1 | 0.029815611 | | 0.73953885 | | GLS |
| path:hsa04934 | Cushing syndrome | | 155 | | 4 | 0.031430973 | | 0.73953885 | | ADCY7,ITPR1,RB1,TCF7 |
| path:hsa05225 | Hepatocellular carcinoma | | 168 | | 4 | 0.03181767 | | 0.73953885 | | LRP6,PRKCB,RB1,TCF7 |
| path:hsa04540 | Gap junction | | 88 | | 3 | 0.031863891 | | 0.73953885 | | ADCY7,ITPR1,PRKCB |
| path:hsa04912 | GnRH signaling pathway | | 93 | | 3 | 0.034721138 | | 0.73953885 | | ADCY7,ITPR1,PRKCB |
| path:hsa04670 | Leukocyte transendothelial migration | | 113 | | 3 | 0.035101182 | | 0.73953885 | | ITGB2,PRKCB,SIPA1 |
| path:hsa04727 | GABAergic synapse | | 89 | | 3 | 0.036441045 | | 0.73953885 | | ADCY7,GLS,PRKCB |
| path:hsa04916 | Melanogenesis | | 101 | | 3 | 0.039042937 | | 0.74832297 | | ADCY7,PRKCB,TCF7 |
| path:hsa04750 | Inflammatory mediator regulation of TRP channels | | 98 | | 3 | 0.04789458 | | 0.82202087 | | ADCY7,ITPR1,PRKCB |
| path:hsa04925 | Aldosterone synthesis and secretion | | 98 | | 3 | 0.049361496 | | 0.82202087 | | ADCY7,ITPR1,PRKCB |
| Enriched GO terms among genes associated with commonly activated probes | |  | |  | | |  | |  | |
|  | ONTOLOGY | | TERM | | N | DE | | P.DE | | FDR |
| GO:0006955 | BP | | immune response | | 1998 | 416 | | 2.30E-20 | | 5.23E-16 |
| GO:0002376 | BP | | immune system process | | 2921 | 589 | | 1.32E-19 | | 1.32E-15 |
| GO:0002682 | BP | | regulation of immune system process | | 1468 | 336 | | 1.74E-19 | | 1.32E-15 |
| GO:0001775 | BP | | cell activation | | 1387 | 329 | | 2.55E-19 | | 1.45E-15 |
| GO:0045321 | BP | | leukocyte activation | | 1230 | 294 | | 6.33E-19 | | 2.42E-15 |
| GO:0046649 | BP | | lymphocyte activation | | 673 | 190 | | 6.40E-19 | | 2.42E-15 |
| GO:0007159 | BP | | leukocyte cell-cell adhesion | | 364 | 120 | | 1.27E-18 | | 4.14E-15 |
| GO:0002694 | BP | | regulation of leukocyte activation | | 516 | 152 | | 4.43E-18 | | 1.16E-14 |
| GO:0042110 | BP | | T cell activation | | 472 | 145 | | 4.60E-18 | | 1.16E-14 |
| GO:0050865 | BP | | regulation of cell activation | | 555 | 160 | | 1.59E-17 | | 3.60E-14 |
| GO:0051249 | BP | | regulation of lymphocyte activation | | 432 | 132 | | 7.69E-17 | | 1.59E-13 |
| GO:0050863 | BP | | regulation of T cell activation | | 325 | 107 | | 2.87E-16 | | 5.44E-13 |
| GO:1903037 | BP | | regulation of leukocyte cell-cell adhesion | | 328 | 105 | | 1.25E-15 | | 2.19E-12 |
| GO:0050776 | BP | | regulation of immune response | | 876 | 210 | | 1.39E-15 | | 2.26E-12 |
| GO:0002252 | BP | | immune effector process | | 1191 | 261 | | 3.45E-14 | | 5.22E-11 |
| GO:0022407 | BP | | regulation of cell-cell adhesion | | 435 | 130 | | 1.07E-13 | | 1.53E-10 |
| GO:0002684 | BP | | positive regulation of immune system process | | 934 | 218 | | 1.14E-13 | | 1.53E-10 |
| GO:0002696 | BP | | positive regulation of leukocyte activation | | 323 | 100 | | 3.40E-13 | | 4.25E-10 |
| GO:0002250 | BP | | adaptive immune response | | 413 | 112 | | 3.55E-13 | | 4.25E-10 |
| GO:0001816 | BP | | cytokine production | | 818 | 185 | | 6.17E-13 | | 7.02E-10 |
| Enriched GO terms among genes associated with infant-specific probes | |  | |  | | |  | |  | |
|  | ONTOLOGY | | TERM | | N | DE | | P.DE | | FDR |
| GO:1902953 | BP | | positive regulation of ER to Golgi vesicle-mediated transport | | 5 | 4 | | 0.0002432 | | 1 |
| GO:0006955 | BP | | immune response | | 1998 | 157 | | 0.00026789 | | 1 |
| GO:0060628 | BP | | regulation of ER to Golgi vesicle-mediated transport | | 15 | 5 | | 0.00122355 | | 1 |
| GO:0035612 | MF | | AP-2 adaptor complex binding | | 9 | 5 | | 0.00169116 | | 1 |
| GO:0033256 | CC | | I-kappaB/NF-kappaB complex | | 4 | 3 | | 0.00253909 | | 1 |
| GO:0050853 | BP | | B cell receptor signaling pathway | | 62 | 12 | | 0.00357795 | | 1 |
| GO:0002376 | BP | | immune system process | | 2921 | 220 | | 0.00416287 | | 1 |
| GO:0001923 | BP | | B-1 B cell differentiation | | 5 | 3 | | 0.00458859 | | 1 |
| GO:0002335 | BP | | mature B cell differentiation | | 31 | 7 | | 0.0046882 | | 1 |
| GO:0050776 | BP | | regulation of immune response | | 876 | 74 | | 0.00476469 | | 1 |
| GO:0071593 | BP | | lymphocyte aggregation | | 5 | 3 | | 0.00551797 | | 1 |
| GO:0005903 | CC | | brush border | | 100 | 16 | | 0.00604279 | | 1 |
| GO:0047696 | MF | | beta-adrenergic receptor kinase activity | | 4 | 3 | | 0.00608568 | | 1 |
| GO:0016598 | BP | | protein arginylation | | 2 | 2 | | 0.00657511 | | 1 |
| GO:0036092 | BP | | phosphatidylinositol-3-phosphate biosynthetic process | | 14 | 5 | | 0.00664325 | | 1 |
| GO:0010986 | BP | | positive regulation of lipoprotein particle clearance | | 10 | 3 | | 0.00694134 | | 1 |
| GO:0050857 | BP | | positive regulation of antigen receptor-mediated signaling pathway | | 23 | 6 | | 0.00696429 | | 1 |
| GO:0004461 | MF | | lactose synthase activity | | 3 | 2 | | 0.00741812 | | 1 |
| GO:2000147 | BP | | positive regulation of cell motility | | 569 | 57 | | 0.00753248 | | 1 |
| GO:0051272 | BP | | positive regulation of cellular component movement | | 581 | 58 | | 0.00822009 | | 1 |
| Enriched GO terms among genes associated with adolescent-specific probes | |  | |  | | |  | |  | |
|  | ONTOLOGY | | TERM | | N | DE | | P.DE | | FDR |
| GO:0001775 | BP | | cell activation | | 1387 | 20 | | 2.56E-05 | | 0.41988195 |
| GO:1903039 | BP | | positive regulation of leukocyte cell-cell adhesion | | 232 | 8 | | 5.01E-05 | | 0.41988195 |
| GO:0046649 | BP | | lymphocyte activation | | 673 | 13 | | 5.90E-05 | | 0.41988195 |
| GO:0006955 | BP | | immune response | | 1998 | 22 | | 7.68E-05 | | 0.41988195 |
| GO:0002682 | BP | | regulation of immune system process | | 1468 | 19 | | 9.57E-05 | | 0.41988195 |
| GO:0007159 | BP | | leukocyte cell-cell adhesion | | 364 | 9 | | 0.00011081 | | 0.41988195 |
| GO:0045321 | BP | | leukocyte activation | | 1230 | 17 | | 0.00013128 | | 0.42638375 |
| GO:0022409 | BP | | positive regulation of cell-cell adhesion | | 274 | 8 | | 0.00021525 | | 0.61172889 |
| GO:1903037 | BP | | regulation of leukocyte cell-cell adhesion | | 328 | 8 | | 0.00029181 | | 0.73718385 |
| GO:0002313 | BP | | mature B cell differentiation involved in immune response | | 26 | 3 | | 0.00037729 | | 0.8578007 |
| GO:0016829 | MF | | lyase activity | | 188 | 6 | | 0.00044575 | | 0.91834711 |
| GO:0002252 | BP | | immune effector process | | 1191 | 15 | | 0.0004922 | | 0.91834711 |
| GO:0030217 | BP | | T cell differentiation | | 246 | 7 | | 0.00052509 | | 0.91834711 |
| GO:0002366 | BP | | leukocyte activation involved in immune response | | 715 | 11 | | 0.00061861 | | 0.92625186 |
| GO:0002250 | BP | | adaptive immune response | | 413 | 8 | | 0.00066046 | | 0.92625186 |
| GO:0002263 | BP | | cell activation involved in immune response | | 719 | 11 | | 0.00068084 | | 0.92625186 |
| GO:0002335 | BP | | mature B cell differentiation | | 31 | 3 | | 0.00071724 | | 0.92625186 |
| GO:0002684 | BP | | positive regulation of immune system process | | 934 | 13 | | 0.00076342 | | 0.92625186 |
| GO:0030098 | BP | | lymphocyte differentiation | | 357 | 8 | | 0.00077405 | | 0.92625186 |
| GO:0002376 | BP | | immune system process | | 2921 | 27 | | 0.00082414 | | 0.93688384 |

**Supplementary table 3:** Table of probes within differentially methylated regions, annotated with ATAC peak overlaps, and proximity to nearest gene, based on GREAT analysis

| Probe_ID | DMR_chromosome | DMR_start | DMR_end | Age_specificity | CTRL_mean_unstim_adolescents | CTRL_mean_stim_adolescents | CTRL_unstim_vs_stim_dB_adolescents | CTRL_mean_unstim_infants | CTRL_mean_stim_infants | CTRL_unstim_vs_stim_dB_infants | Relation_to_Island.y | GencodeBasicV12_NAME.y | GencodeBasicV12_Accession.y | GencodeBasicV12_Group.y | GencodeCompV12_NAME.y | DNase_Hypersensitivity_NAME.y | Relation_to_Island2 | Dynamic_chromatin |
| --- | --- | --- | --- | --- | --- | --- | --- | --- | --- | --- | --- | --- | --- | --- | --- | --- | --- | --- |
| cg00005599 | chr14 | 22974144 | 22975521 | Significant_in_both | 0.46984518 | 0.28290571 | -0.1869395 | 0.79509961 | 0.57308874 | -0.2220109 | OpenSea | TRAJ35 | ENST00000390502.1 | TSS1500 | TRAJ35 |  | OpenSea | No dynamic peak present |
| cg00006081 | chr10 | 124908427 | 124910812 | Significant_in_adolescents | 0.25912537 | 0.31059823 | 0.05147286 | 0.21077013 | 0.24196721 | 0.03119708 | Island |  |  |  |  | chr10:124908880-124910015 | Island | No dynamic peak present |
| cg00006301 | chr10 | 62332794 | 62334454 | Significant_in_adolescents | 0.21770372 | 0.17365614 | -0.0440476 | 0.34206473 | 0.29045323 | -0.0516115 | OpenSea | ANK3;ANK3;ANK3 | ENST00000373817.4;ENST00000503366.1;ENST00000510382.1 | TSS1500;TSS1500;3'UTR | ANK3;ANK3;ANK3;ANK3 | chr10:62332260-62332955 | OpenSea | No dynamic peak present |
| cg00007076 | chr8 | 67342485 | 67343633 | Significant_in_adolescents | 0.70275134 | 0.59453664 | -0.1082147 | 0.71215617 | 0.57271371 | -0.1394425 | N_Shore | RRS1;RP11-346I3.4;RRS1 | ENST00000320270.2;ENST00000499642.1;ENST00000320270.2 | 1stExon;TSS1500;3'UTR | RRS1;RP11-346I3.4;ADHFE1;RRS1;ADHFE1 | chr8:67341365-67342710 | Shore | No dynamic peak present |
| cg00030290 | chr2 | 8716921 | 8717199 | Significant_in_both | 0.469666 | 0.37100882 | -0.0986572 | 0.54131744 | 0.42554496 | -0.1157725 | OpenSea | AC011747.4;AC011747.4 | ENST00000436187.1;ENST00000454224.1 | TSS200;3'UTR | AC011747.4;AC011747.4 | chr2:8716960-8717315 | OpenSea | No dynamic peak present |
| cg00032366 | chr19 | 5691122 | 5692622 | Significant_in_infants | 0.7559514 | 0.70374114 | -0.0522103 | 0.81497851 | 0.74723365 | -0.0677449 | Island |  |  |  |  | chr19:5690400-5692715 | Island | No dynamic peak present |
| cg00073460 | chr6 | 149805292 | 149806659 | Significant_in_infants | 0.56690019 | 0.53155982 | -0.0353404 | 0.67807912 | 0.56756196 | -0.1105172 | OpenSea | ZC3H12D;ZC3H12D;ZC3H12D | ENST00000416573.2;ENST00000542614.1;ENST00000409806.3 | TSS1500;TSS1500;TSS1500 | ZC3H12D;ZC3H12D;ZC3H12D | chr6:149805825-149806855 | OpenSea | No dynamic peak present |
| cg00078299 | chr8 | 124284176 | 124285055 | Significant_in_both | 0.73682071 | 0.39967171 | -0.337149 | 0.82294299 | 0.38632017 | -0.4366228 | N_Shore | ZHX1;ZHX1;ZHX1;ZHX1 | ENST00000522595.1;ENST00000297857.2;ENST00000395571.2;ENST00000522655.1 | 3'UTR;5'UTR;5'UTR;5'UTR | ZHX1;ZHX1;ZHX1;ZHX1;ZHX1;ZHX1 | chr8:124284080-124284535 | Shore | No dynamic peak present |
| cg00082664 | chr4 | 154710224 | 154712902 | Significant_in_adolescents | 0.04697502 | 0.04818903 | 0.00121401 | 0.06685816 | 0.06449268 | -0.0023655 | Island | SFRP2 | ENST00000274063.4 | TSS1500 | SFRP2 | chr4:154710720-154711935 | Island | No dynamic peak present |
| cg00088264 | chr8 | 103136760 | 103137351 | Significant_in_adolescents | 0.15164876 | 0.1511085 | -0.0005403 | 0.2365915 | 0.23309851 | -0.003493 | Island | NCALD;NCALD;NCALD | ENST00000311028.3;ENST00000521599.1;ENST00000395923.1 | TSS200;TSS1500;5'UTR | NCALD;NCALD;NCALD;NCALD;NCALD;NCALD;NCALD;NCALD;NCALD;NCALD | chr8:103134465-103137015 | Island | No dynamic peak present |
| cg00103398 | chr1 | 173834077 | 173836727 | Significant_in_both | 0.71265386 | 0.69625776 | -0.0163961 | 0.76773342 | 0.71416491 | -0.0535685 | N_Shelf | GAS5;SNORD81;SNORD47;SNORD80 | ENST00000431268.1;ENST00000363840.1;ENST00000365524.1;ENST00000364822.1 | 3'UTR;TSS1500;TSS1500;TSS200 | GAS5;GAS5;GAS5;GAS5;GAS5;GAS5;GAS5;GAS5;GAS5;GAS5;GAS5;GAS5;SNORD81;GAS5;GAS5;SNORD47;GAS5;GAS5;GAS5 | chr1:173834000-173834450 | Shelf | No dynamic peak present |
| cg00103549 | chr14 | 22945102 | 22948498 | Significant_in_both | 0.76233446 | 0.66340701 | -0.0989275 | 0.84075167 | 0.72606539 | -0.1146863 | OpenSea | TRAJ58;TRAJ59 | ENST00000390481.1;ENST00000390480.2 | TSS1500;TSS200 | TRAJ58;TRAJ59;AE000661.37;AE000661.37 | chr14:22945320-22945530 | OpenSea | No dynamic peak present |
| cg00188443 | chr14 | 23006993 | 23010822 | Significant_in_infants | 0.12431772 | 0.12546053 | 0.0011428 | 0.17992208 | 0.13552239 | -0.0443997 | OpenSea | TRAJ4 | ENST00000390533.1 | TSS1500 | TRAJ4 |  | OpenSea | Dynamic peak present |
| cg00190116 | chr14 | 22986466 | 22990203 | Significant_in_both | 0.28853226 | 0.24860319 | -0.0399291 | 0.42242937 | 0.29325963 | -0.1291697 | OpenSea | TRAJ26;TRAJ25 | ENST00000390511.1;ENST00000390512.2 | TSS1500;TSS1500 | TRAJ26;TRAJ25 | chr14:22986160-22986570 | OpenSea | No dynamic peak present |
| cg00194126 | chr2 | 157182707 | 157186681 | Significant_in_infants | 0.12422621 | 0.12780785 | 0.00358164 | 0.16749556 | 0.1664032 | -0.0010924 | Island |  |  |  |  | chr2:157185900-157187435 | Island | No dynamic peak present |
| cg00207731 | chr15 | 91415532 | 91416118 | Significant_in_adolescents | 0.73188831 | 0.57416824 | -0.1577201 | 0.81425043 | 0.67153315 | -0.1427173 | S_Shore | FURIN | ENST00000268171.3 | 5'UTR | FURIN;FURIN;FURIN | chr15:91415840-91416135 | Shore | No dynamic peak present |
| cg00209129 | chr8 | 134261857 | 134262129 | Significant_in_both | 0.77208276 | 0.64320858 | -0.1288742 | 0.80947806 | 0.63552526 | -0.1739528 | OpenSea | NDRG1 | ENST00000521414.1 | TSS200 | NDRG1;NDRG1;NDRG1;NDRG1;NDRG1;NDRG1;NDRG1 | chr8:134261565-134262030 | OpenSea | No dynamic peak present |
| cg00231528 | chr1 | 9714280 | 9714845 | Significant_in_infants | 0.04331371 | 0.05170886 | 0.00839515 | 0.05576568 | 0.05536441 | -0.0004013 | S_Shore | C1orf200;PIK3CD;PIK3CD;PIK3CD | ENST00000377320.3;ENST00000536656.1;ENST00000377346.4;ENST00000469267.1 | 1stExon;5'UTR;5'UTR;5'UTR | C1orf200;PIK3CD;PIK3CD;PIK3CD | chr1:9713545-9714795 | Shore | No dynamic peak present |
| cg00234176 | chr5 | 156642345 | 156644040 | Significant_in_infants | 0.87057692 | 0.77384179 | -0.0967351 | 0.90762158 | 0.84568936 | -0.0619322 | OpenSea | CTB-4E7.1 | ENST00000519375.1 | 3'UTR | ITK;CTB-4E7.1 | chr5:156642865-156643115 | OpenSea | No dynamic peak present |
| cg00240195 | chr2 | 157182707 | 157186681 | Significant_in_infants | 0.67870118 | 0.74871704 | 0.07001586 | 0.61522699 | 0.73527871 | 0.12005173 | N_Shore |  |  |  |  | chr2:157184505-157185155 | Shore | No dynamic peak present |
| cg00245098 | chr14 | 22982238 | 22983224 | Significant_in_both | 0.82255731 | 0.77955094 | -0.0430064 | 0.87364785 | 0.81245617 | -0.0611917 | OpenSea | TRAJ28 | ENST00000390509.1 | TSS1500 | TRAJ28 |  | OpenSea | No dynamic peak present |
| cg00308563 | chr2 | 106473189 | 106474173 | Significant_in_infants | 0.11911808 | 0.0973666 | -0.0217515 | 0.1507636 | 0.10005625 | -0.0507074 | OpenSea | AC009505.2 | ENST00000427050.1 | 3'UTR | AC009505.2 | chr2:106472900-106473410 | OpenSea | No dynamic peak present |
| cg00312486 | chr1 | 209876837 | 209878389 | Significant_in_both | 0.63873059 | 0.44804787 | -0.1906827 | 0.77417664 | 0.52140899 | -0.2527676 | OpenSea | HSD11B1;HSD11B1;HSD11B1;RP1-28O10.1 | ENST00000367027.3;ENST00000367028.1;ENST00000261465.1;ENST00000441672.1 | TSS1500;5'UTR;5'UTR;3'UTR | HSD11B1;HSD11B1;HSD11B1;RP1-28O10.1 | chr1:209877265-209877510 | OpenSea | No dynamic peak present |
| cg00313981 | chr1 | 228595062 | 228596966 | Significant_in_infants | 0.23704237 | 0.21998314 | -0.0170592 | 0.2532538 | 0.22920926 | -0.0240445 | S_Shore | TRIM11;TRIM11;RP11-245P10.4 | ENST00000366699.3;ENST00000284551.6;ENST00000436779.1 | TSS1500;TSS1500;TSS200 | TRIM11;TRIM11;TRIM11;RP11-245P10.4 | chr1:228594920-228595215 | Shore | No dynamic peak present |
| cg00314247 | chr17 | 76408337 | 76410298 | Significant_in_infants | 0.87195161 | 0.86569861 | -0.006253 | 0.89284403 | 0.89139312 | -0.0014509 | OpenSea | PGS1 | ENST00000335081.7 | 3'UTR | PGS1 |  | OpenSea | No dynamic peak present |
| cg00322767 | chr12 | 9820221 | 9822287 | Significant_in_both | 0.15058946 | 0.10043321 | -0.0501563 | 0.18890728 | 0.13763283 | -0.0512745 | OpenSea | CLEC2D;CLEC2D;CLEC2D | ENST00000261340.7;ENST00000543300.1;ENST00000487752.1 | TSS1500;TSS1500;5'UTR | CLEC2D;CLEC2D;CLEC2D;CLEC2D;CLEC2D;CLEC2D;CLEC2D;CLEC2D | chr12:9821520-9821790 | OpenSea | No dynamic peak present |

**Abbreviated table, please contact corresponding author for full table.

**Supplementary table 4:** Raw cytokine measurements of all infants and adolescents assessed in this study

| ID | Age | IFN-γ | IL-2 | IL-6 | IL-10 | TNF |
| --- | --- | --- | --- | --- | --- | --- |
| X2202_Activated | Adolescent | 26.18 | 13478.46 | 18.3 | 512.05 | 458.59 |
| X2202_Quiescent | Adolescent | 0.4 | 10355.55 | 0.8 | 0.68 | 0.6 |
| X3752_Activated | Adolescent | 4.2 | 11616.52 | 9.22 | 32.76 | 236.54 |
| X3752_Quiescent | Adolescent | 0.4 | 8319.42 | 4.06 | 0.55 | 0.6 |
| X3762_Activated | Adolescent | 413.8 | 11227.51 | 4.52 | 214.58 | 786.87 |
| X3762_Quiescent | Adolescent | 0.4 | 8379.52 | 0.8 | 0.06 | 0.6 |
| X3874_Activated | Adolescent | 232.12 | 9764.39 | 4.45 | 128.69 | 560.46 |
| X3874_Quiescent | Adolescent | 0.4 | 6645.19 | 0.8 | 0.09 | 0.6 |
| X4219_Activated | Adolescent | 74.32 | 14888.21 | 40.03 | 65.35 | 500.72 |
| X4219_Quiescent | Adolescent | 0.4 | 8817.26 | 0.8 | 0.06 | 0.6 |
| X4502_Activated | Adolescent | 84.69 | 11626.52 | 8.16 | 268.94 | 460.07 |
| X4502_Quiescent | Adolescent | 0.4 | 7684.7 | 0.8 | 0.22 | 0.6 |
| X4730_Activated | Adolescent | 14.77 | 9768.26 | 2.12 | 97.2 | 264.45 |
| X4730_Quiescent | Adolescent | 0.4 | 6528.46 | 0.8 | 0.64 | 0.6 |
| X5603_Activated | Adolescent | 26.1 | 11878.87 | 2.58 | 73.32 | 439.48 |
| X5603_Quiescent | Adolescent | 0.4 | 9068.52 | 0.8 | 0.06 | 0.6 |
| X5827_Activated | Adolescent | 14.46 | 12107.85 | 4.05 | 104.97 | 203.71 |
| X5827_Quiescent | Adolescent | 0.4 | 8525.54 | 0.8 | 0.24 | 0.6 |
| X6341_Activated | Adolescent | 12.55 | 10364.31 | 7.65 | 67.31 | 273.47 |
| X6341_Quiescent | Adolescent | 0.4 | 4203.78 | 0.8 | 0.06 | 0.6 |
| X6398_Activated | Adolescent | 14.85 | 8125.47 | 5.6 | 180.26 | 276.21 |
| X6398_Quiescent | Adolescent | 0.4 | 4444.05 | 0.8 | 0.06 | 0.6 |
| X7034_Activated | Adolescent | 119.41 | 14244.4 | 79.61 | 62.21 | 637.76 |
| X7034_Quiescent | Adolescent | 0.4 | 7683.12 | 0.8 | 0.06 | 0.6 |
| X7076_Activated | Adolescent | 215.23 | 10442.02 | 23.13 | 122.79 | 383.26 |
| X7076_Quiescent | Adolescent | 0.4 | 4633.35 | 0.8 | 0.06 | 0.6 |
| X7346_Activated | Adolescent | 31.4 | 9906.82 | 8.47 | 24.66 | 208.09 |
| X7346_Quiescent | Adolescent | 0.4 | 9074.07 | 8.68 | 1 | 0.6 |
| X7414_Activated | Adolescent | 43.32 | 6936.6 | 4.1 | 150.85 | 307.66 |
| X7414_Quiescent | Adolescent | 0.4 | 3395.33 | 0.8 | 0.06 | 0.6 |
| X7524_Activated | Adolescent | 275.66 | 12381.55 | 10.38 | 78.32 | 283.01 |
| X7524_Quiescent | Adolescent | 0.4 | 8171.09 | 0.8 | 0.06 | 0.6 |
| X8977_Activated | Adolescent | 21.59 | 13246.18 | 9.84 | 107.77 | 494.95 |
| X8977_Quiescent | Adolescent | 0.4 | 4569.13 | 0.8 | 0.06 | 0.6 |

***Abbreviated table, please contact corresponding author for full table.

**Supplementary table 5:** Results of functional enrichment analysis (in KEGG databases) of genes linked to age-associated probes that overlapped with previous T-cell study (Dozmorov et al.)

|  | Description | N | DE | P.DE | FDR | SigGenesInSet |
| --- | --- | --- | --- | --- | --- | --- |
| path:hsa04064 | NF-kappa B signaling pathway | 103 | 5 | 0.00063061 | 0.21756208 | LAT,BCL2,TRAF1,ZAP70,MAP3K14 |
| path:hsa04650 | Natural killer cell mediated cytotoxicity | 124 | 4 | 0.00741163 | 0.87110722 | LAT,KLRD1,PIK3CD,ZAP70 |
| path:hsa04660 | T cell receptor signaling pathway | 102 | 4 | 0.00863794 | 0.87110722 | LAT,PIK3CD,ZAP70,MAP3K14 |
| path:hsa05162 | Measles | 139 | 4 | 0.01107502 | 0.87110722 | OAS2,PIK3CD,BCL2,CCND3 |
| path:hsa05169 | Epstein-Barr virus infection | 199 | 5 | 0.01271738 | 0.87110722 | OAS2,PIK3CD,BCL2,CCND3,MAP3K14 |
| path:hsa04210 | Apoptosis | 135 | 4 | 0.01514969 | 0.87110722 | CTSD,PIK3CD,BCL2,TRAF1,MAP3K14 |
| path:hsa05235 | PD-L1 expression and PD-1 checkpoint pathway in cancer | 89 | 3 | 0.02948421 | 1 | LAT,PIK3CD,ZAP70 |
| path:hsa00513 | Various types of N-glycan biosynthesis | 39 | 2 | 0.03478363 | 1 | B4GALT1,MAN1C1 |
| path:hsa05222 | Small cell lung cancer | 92 | 3 | 0.04088784 | 1 | PIK3CD,BCL2,TRAF1 |
| path:hsa04668 | TNF signaling pathway | 112 | 3 | 0.04284786 | 1 | PIK3CD,TRAF1,MAP3K14 |
| path:hsa00510 | N-Glycan biosynthesis | 50 | 2 | 0.04744634 | 1 | B4GALT1,MAN1C1 |
| path:hsa05203 | Viral carcinogenesis | 193 | 4 | 0.04872817 | 1 | PIK3CD,TRAF1,CCND3,HDAC4 |
| path:hsa04066 | HIF-1 signaling pathway | 109 | 3 | 0.05064215 | 1 | EGLN2,PIK3CD,BCL2 |
| path:hsa05417 | Lipid and atherosclerosis | 215 | 4 | 0.05295678 | 1 | MIB2,NCF4,PIK3CD,BCL2 |
| path:hsa04380 | Osteoclast differentiation | 127 | 3 | 0.05846604 | 1 | NCF4,PIK3CD,MAP3K14 |
| path:hsa04630 | JAK-STAT signaling pathway | 158 | 3 | 0.0667402 | 1 | LEPR,PIK3CD,BCL2,CCND3 |
| path:hsa04071 | Sphingolipid signaling pathway | 118 | 3 | 0.07085122 | 1 | CTSD,S1PR1,PIK3CD,BCL2 |
| path:hsa05206 | MicroRNAs in cancer | 309 | 4 | 0.07189653 | 1 | DNMT3B,PIK3CD,BCL2,HDAC4 |
| path:hsa04613 | Neutrophil extracellular trap formation | 175 | 3 | 0.07463601 | 1 | NCF4,PIK3CD,HDAC4 |
| path:hsa05164 | Influenza A | 167 | 3 | 0.07797628 | 1 | OAS2,PIK3CD,CCND3 |
